# Study of Sorption Kinetics and Sorption–Desorption Models to Assess the Transport Mechanisms of 2,4-Dichlorophenoxyacetic Acid on Volcanic Soils

**DOI:** 10.3390/ijerph18126264

**Published:** 2021-06-09

**Authors:** Lizethly Cáceres-Jensen, Jorge Rodríguez-Becerra, Carlos Garrido, Mauricio Escudey, Lorena Barrientos, Jocelyn Parra-Rivero, Valentina Domínguez-Vera, Bruno Loch-Arellano

**Affiliations:** 1 Laboratorio de Fisicoquímica & Analítica (PachemLab), Departamento de Química, Facultad de Ciencias Básicas, Universidad Metropolitana de Ciencias de la Educación, Santiago 7760197, Chile; jorge.rodriguez@umce.cl (J.R.-B.); joselyn.parrarivero@gmail.com (J.P.-R.); valentina.dominguez@umce.cl (V.D.-V.); loch.arellano@gmail.com (B.L.-A.); 2 Laboratorio de Química Inorgánica, Departamento de Química, Facultad de Ciencias Básicas, Universidad Metropolitana de Ciencias de la Educación, Santiago 7760197, Chile; carlos.garrido@umce.cl; 3 Facultad de Química y Biología, Universidad de Santiago de Chile, Santiago 9170020, Chile; mauricio.escudey@usach.cl; 4 Center for the Development of Nanoscience and Nanotechnology (CEDENNA), Santiago 9170020, Chile; 5 Centro de Investigación en Nanotecnología y Materiales Avanzados (CIEN-UC), Facultad de Química, Pontificia Universidad Católica de Chile, Santiago 7820436, Chile; lbarrientop@uc.cl; 6 Millenium Nuclei on Catalytic Processes towards Sustainable Chemistry (CSC), Santiago 7820436, Chile; 7 Facultad de Ciencias, Universidad de Chile, Santiago 7800003, Chile

**Keywords:** herbicides, variable-charge-soils, sorption mechanism, sorption kinetics models, spectroscopic analysis

## Abstract

The sorption behavior of 2,4-dichlorophenoxyacetic acid (2,4-D) in the abundant agricultural volcanic ash-derived soils (VADS) is not well understood despite being widely used throughout the world, causing effects to the environment and human health. The environmental behavior and risk assessment of groundwater pollution by pesticides can be evaluated through kinetic models. This study evaluated the sorption kinetics and 2,4-D sorption–desorption in ten VADS through batch sorption experiments. Differences in the sorption extent for the fast and slow phases was observed through the IPD model where 2,4-D sorption kinetics was controlled by external mass transfer and intra organic matter diffusion in Andisols ($C_{1}$ ≠ 0). We confirmed from the spectroscopic analysis that the carboxylate group directly drives the interaction of 2,4-D on Andisol soil. The MLR model showed that IEP, $Fe_{DCB}$, and pH×Silt are important soil descriptors in the 2,4-D sorption in VADS. The Freundlich model accurately represented sorption equilibrium data in all cases ($K_{f}$ values between 1.1 and 24.1 µg^1−1/n^ mL^1/n^g^−1^) with comparatively higher sorption capacity on Andisols, where the highest hysteresis was observed in soils that presented the highest and lowest OC content (H close to 0).

## 1. Introduction

Herbicide contamination of surface and groundwater is a major concern due to the widespread use of these chemicals in agricultural and urban areas and the decline in biodiversity [1]. 2,4-Dichlorophenoxyacetic acid (2,4-D) is a selective, post-emergence ionizable herbicide developed by the Dow company, corresponding to a formulation broadly used widely around the world to control annual and perennial herbs in pastures, fruits, cereals, hay, wheat, maize, barley cultivation, sorghum, sugar cane, and rice [2,3,4]. The relative persistence and mobility of the said herbicide are of global public concern over the potential of 2,4-D and its primary metabolites to contaminate groundwater [4] and the effects at the ecosystem level because rice production relies heavily on ample water supply [5]. The World Health Organization has classified 2,4-D as “probably carcinogenic to humans”. The highest level of 2,4-D allowed in drinking water is 0.07 mg L^−1^ [6]. The potential health effects from ingestion of water range from physiological abnormalities and carcinogenesis.

The leaching of 2,4-D has been documented from soil to water sources due to its high use, persistence, polarity, and low sorption [2,3]. In this regard, 2,4-D sorption to soil may significantly affect the environment’s eventual fate [4]. Hence, it is important to elucidate the kind of interaction of 2,4-D with the soil, where the risk of groundwater contamination with 2,4-D in different types of soil could vary. Furthermore, the soil physicochemical properties and the forms of 2,4-D found in most soils ($pH_{soil}$ between 4 and 8) due to its acidic carboxyl group ($pK_{a}$ = 2.8) will mediate the soil interactions of the said herbicide based on different biogeochemical and ecosystem processes.

Volcanic ash-derived soils (VADS) are relevant in economies relying on forestry and agricultural exports [7,8,9,10,11,12] of countries in Asia, Africa, Oceania, and America [7,8]. The VADS in Chile accounts for 69% of its arable land [13,14,15,16], where agriculture is developed in the central region (from 19° to 56° S latitude). Among the VADS, Andisols and Ultisols are the most abundant in that region; at the same time, the Chilean National Agriculture and Livestock Services (SAG) indicates that 2,4-D is one of the most widely sold herbicides in that country [17]. Therefore, understanding the behavior 2,4-D in VADS and its potential risk of leaching into groundwater is essential for human health [18]. Furthermore, it is vital to be able to timely evaluate their sensitivity to anthropogenic alterations such as the use of herbicides [19,20], given the economic value of these soils of high quality.

Time-dependent sorption (or non-ideal sorption) can result from physical or chemical non-equilibrium [21]. Regarding the ionizable herbicide sorption behavior in VADS, sorption kinetic is a non-equilibrium process [1,12,21,22,23]. In general, non-equilibrium sorption has been attributed to diffusive mass transfer (MT) resistances, non-linearity in sorption isotherms, positive hysteresis, and rate-limited sorption reactions [21]. The intra organic matter diffusion (IOMD) has been suggested to be the predominant factor responsible for the chemical non-equilibrium sorption of non-ionic or hydrophobic compounds and it can occur during pesticide transport in soils [24].

Considering the widespread use of 2,4-D in agriculture, its effects on human health and the scarce reports on 2,4-D sorption behavior in VADS, the aims of this work were (i) to establish 2,4-D sorption kinetics in ten different agricultural VADS from Chile; (ii) to apply solute transport mechanism models involved in 2,4-D sorption on VADS; (iii) to establish the 2,4-D sorption–desorption on VADS; (iv) to explore possible 2,4-D sorption mechanisms in VADS by mean of spectroscopic analysis; and (v) to identify important soil descriptors in the 2,4-D sorption on VADS by means of the MLR model. The fulfilment of these aims serves to elucidate the 2,4-D environmental behavior and the potential environmental consequences.

## 2. Materials and Methods

### 2.1. Soil Samples

The VADS were collected from the 0 to 15-cm layer in southern-central Chile’s agricultural regions (Table 1). The Walkley–Black method was used to determine the soil OC content [25]. The Blake method was used to determine the cation exchange capacity (CEC) [26]. pH was measured in soil suspensions with a soil to water ratio of 1:2.5 (*w*/*v*). The isoelectric point (IEP) was determined by means of electrophoretic measurements. The mineralogy and chemical composition of these soils have been previously described [27].

### 2.2. Analytical methods

The quantitative analysis of 2,4-D was conducted by HPLC-DAD (Shimadzu, Kyoto, Japan) (Table 2). Quality parameters for the chromatographic analysis were previously established. Analytical sensitivity, detection, and quantification limits were 0.003, 0.009, and 0.032 μg mL^−1^, respectively (Table 2). These values were calculated from a calibration curve carried out at eight concentration levels: 0.025, 0.050, 0.075, 0.100, 0.250, 0.500, 0.750, and 1.000 μg mL^−1^. The chromatographic response was found to be linear in this concentration range with an $R^{2}$ value of 0.999. The data of 2,4-D in solutions were analyzed using SigmaPlot V13 (Systat Software, Inc., San Jose, CA, USA). The Surface-Enhanced Raman Scattering (SERS) measurements were performed using a Renishaw micro-Raman RM 1000 spectrometer, equipped with laser lines 514, 633, and 785 nm. The spectrometer was coupled to a Leica microscope DMLM (Renishaw, Gloucestershire, UK), and a CCD camera electrically cooled. The Raman signal was calibrated to the 520 cm^−1^ line of silicon and lens of 50× objective. The laser power on the sample was about 0.2 mW. Acquisition time was set between 10 and 20 s per accumulation; the average of accumulations was 5 with a spectral resolution of 4 cm^−1^. The spectra were recorded between 100 and 1800 cm^−1^. Spectral recording conditions and the laser line’s choice to be used were selected to avoid degradation of the sample; in this sense, the 633 and 785 nm laser lines were used.

### 2.3. Chemicals

All reagents used were analytical or HPLC grade. All of these chemicals were used as received, and aqueous stock solutions of chemicals were made in ultrapure water (conductivity 0.05 µS cm^−1^). The analytical reference standard, 2,4-D (99.7% purity; Sigma-Aldrich) was used for the preparation of the stock solution of 1 mg mL^−1^ in acetonitrile (AcN) (Table 2). For the SERS measurements, the analyte’s aqueous stock solutions were prepared in ultrapure water to a final concentration of 3.6 × 10^−3^ M. A low charged Ag colloidal solution was used [28]. For colloidal solutions of silver nanoparticles (AgNps), we used silver nitrate 99.9999% trace metals basis (product number 204390; Sigma-Aldrich, St. Louis, MO, USA), sodium hydroxide (product number 106469, EMSURE^®^ ACS, Reag. Ph Eur; Merck, Darmstadt, Germany), and hydroxylamine hydrochloride 99.999% trace metals basis (product number 431362; Sigma-Aldrich, St. Louis, MO, USA).

### 2.4. Sorption Kinetic Experiments, Sorption–Desorption Experiments and Models

#### 2.4.1. Kinetic Sorption Experiments

Kinetic experiments were carried out at 25 ± 1 °C. Duplicate samples of 2 g of air-dried soils were mixed with a 10 mL aliquot of an aqueous solution of 5 μg mL^−1^ 2,4-D (in 0.01 M CaCl_2_) in 50 mL centrifuge tubes. The tubes were shaken “end over end” at the natural pH of soils during 5, 15, 30, 45, 60, 90, 120, and 180 min and then centrifuged at 3250 rpm for 20 min. Each supernatant was filtered through a 0.22 μm membrane pore-size Millipore filter. The concentration of 2,4-D in solutions was determined by HPLC-DAD (Table 2). Interferences from soils were discarded through the corresponding purity and matching tests. Table 3 shows the theoretical and empirical description of each sorption kinetics model.

#### 2.4.2. Sorption–Desorption Experiments

All isotherm experiments were carried out at 25 ± 1 °C. Duplicate samples of 2 g air-dried soils were mixed with a 10 mL aliquot of aqueous solutions of 2,4-D at 5, 10, 15, 20 and 25 μg mL^−1^ (in 0.01 M CaCl_2_) in 50 mL centrifuge tubes. The tubes were shaken end-over-end at natural pH of soils for 24 h to ensure equilibrium and then centrifuged at 3200 rpm for 20 min. The supernatant was filtered through a 0.22 μm pore-size Millipore filter. Desorption was performed using the samples treated at 25 μg mL^−1^. After the sorption equilibrium was reached, 5 mL of the supernatant solution was replaced with 5 mL of herbicide-free 0.01 M CaCl_2_ solution, and samples were shaken again for 2 h, followed by centrifugation. The same step was repeated four consecutive times; every time, an aliquot of the centrifuged supernatant was removed for analysis. The final concentration of 2,4-D in solution was determined by HPLC-DAD with the same analytical methods indicated previously in kinetics studies (Table 2). The amount of 2,4-D adsorbed was calculated from Equation (1) (Table 3). Table 3 shows the theoretical and empirical description of each sorption model.

### 2.5. Sample preparation for the Raman and SERS Measurements

Raman measurements were performed for the 2,4-D crystals deposited on a quartz slide. To evaluate the Andisol soil (NBR) interaction with 2,4-D, the following methodology was used: 0.0087 g of soil was added to 500 µL of ultrapure water, and 10 µL of 3.6 × 10^−3^ M aqueous solution of 2,4-D was added. The final concentration of 2,4-D in the sample was close to 7.0 × 10^−5^ M. The above mixture was stirred for 20 min and allowed to stand for 24 h at room temperature, and then 500 μL of silver colloid was added and stirred for 20 min. Finally, the sample was centrifuged at 4000 rpm, the supernatant was removed, and then 5 μL of the soil-AgNps sample was taken with a micropipette, and the SERS spectrum was recorded [31].

### 2.6. Statistical Analysis

Pearson’s correlation matrix was used to identify the most correlated variables between VADS properties and 2,4-D sorption coefficient. The “psych” and “corrplot” R packages were employed to perform the correlation analysis, correlogram as well as to assess the potential of multiple linear regression (MLR) models as exploratory modeling. The study was started with 40 soil descriptors or pairwise soil interactions selected to represent different sources of physiochemical information of the VADS regarding their properties like texture, charge, soil constituents, and pH (Table 4). The inter-descriptor correlation coefficient used was less than $R^{2}$ < 0.6 to avoid descriptors being intercorrelated. The generated dataset was used in an automated soil descriptors selection procedure using the best subset and stepwise model selection procedures. These methods were used to reduce data dimensionality and determine if a complex model (more soil descriptors) was significantly better than a less complex model. In this regard, $K_{f}$ values for each soil were modeled using VADS properties individually, and the pairwise products of texture soil properties and soil constituents (e.g., Sand×Clay or $Fe_{Pyro}\times Fe_{DCB}$) to begin to account for interactions between these variables. Additionally, texture soil properties normalized by OC content were included.

The pairwise interactions chosen in the MLR analysis were based on the previously presented antecedents of physical properties (e.g., pH and texture interactions moderate the surface charge of the minerals but may also impact the net charge of the 2,4-D molecule).

(16) $$K_{f}=c+\sum_{i=1}^{n}s_{i}S_{i}+p_{i}P_{i}$$

In Equation (16), $s_{i}$ and $p_{i}$ are coefficients related to the properties of VADS individually and pairwise, respectively. Models were compared through various statistical tests; a high F-statistic value was used combined with the *p*-value < 0.05 to assess the regression model’s significance. $R_{adj}^{2}$ was used as a measure of the goodness of fit.

## 3. Results and Discussion

### 3.1. Physiochemical Properties of VADS

The soils studied presented acidic pH (4.1–5.7, Table 4). 2,4-D is a phenoxyacetic acid with ionic equilibrium constants related to the acidic carboxyl group ($pK_{a}$ = 2.97, Table 2). 2,4-D is in the anionic form according to its $pK_{a}$ values (Table 2) and may be relatively mobile in aqueous systems. All soils presented a negative net charge (IEP value lower than its $pH_{H_{2}O}$) (Table 4). Ultisol soils presented the lowest OC content and IEP than Andisols and a higher clay mineral per cent (Table 4).

### 3.2. Sorption Kinetics

**Figure 1:**
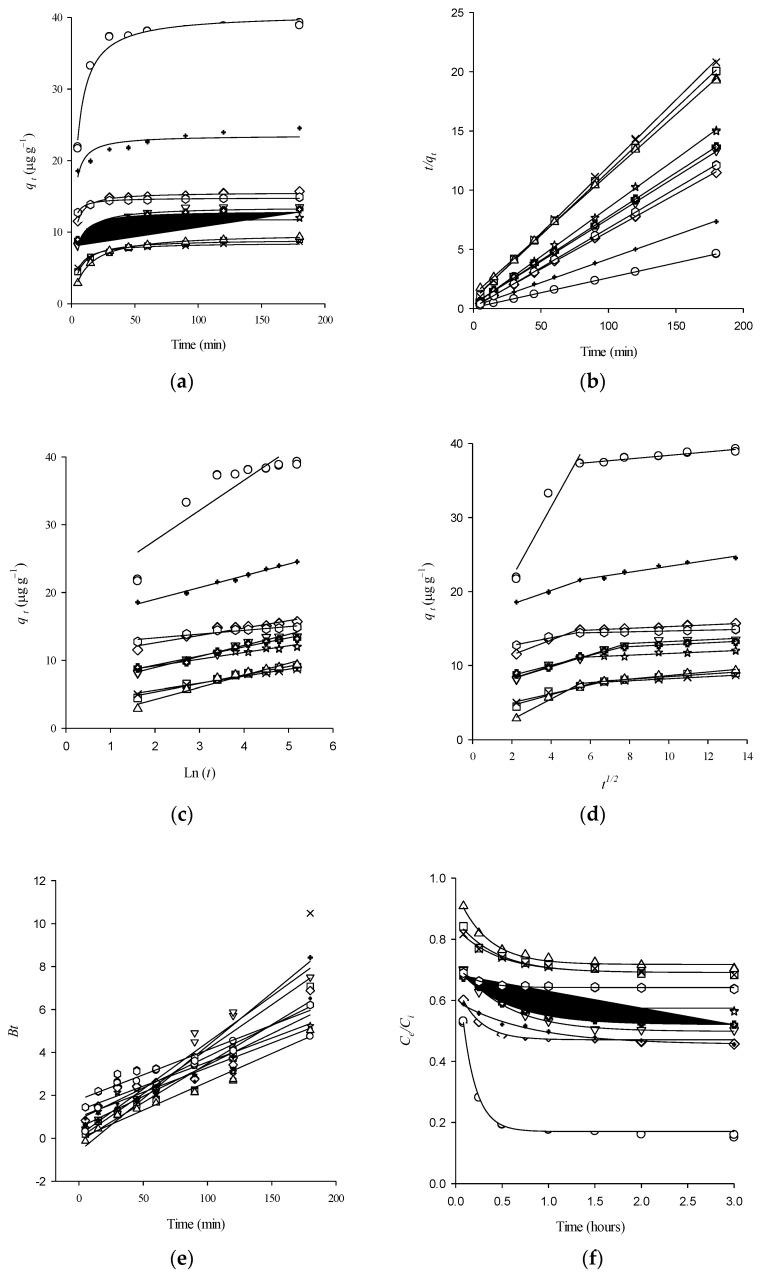
Plot for 2,4-D sorption on volcanic ash-derived soils: COLL (□), MET (×), FRE (☆), STB (◇), OSN (▽), DIG (△), TEM (+), RAL (+), NBR (○) and FRU (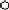). (**a**) Hyperbolic; (**b**) Pseudo-second-order (PSO); (**c**) Elovich; (**d**) Intraparticle Diffusion (IPD); (**e**) Boyd, and (**f**) Two-Site Non-Equilibrium (TSNE) models. Symbols represent the experimental data, and lines represent the theoretical curves described by each model.

The amount of 2,4-D adsorbed per unit mass of the adsorbent increased quickly during the first 10 min in all VADS, followed by slower progress toward equilibrium (Figure 1a).

#### Pseudo-First-Order (PFO) and Pseudo-Second-Order (PSO) Models

Table 3 shows the theoretical and empirical descriptions for each sorption kinetic model. The PSO model correctly described 2,4-D sorption data at all-time intervals ($R^{2}$ > 0.9992; Table S1, Figure S1a). The $q_{max}$ values estimated by PSO model agreed with the experimental data and yielded suitable SE for $q_{max}$ and $k_{2}$ values (Table 5, Figure 1b). 2,4-D showed the slowest sorption on RAL and NBR soils (highest OC content; Table 4) and DIG soil (intermediate OC content; Table 4). For Ultisols, the differences were more pronounced in the early stages of 2,4-D sorption, where the lowest h and highest $t_{1/2}$ values were observed in Ultisols and DIG soil (Table 5). A higher $q_{max}$ of 2,4-D was observed in NBR soil (highest OC and silt content, Table 4). For Andisols, OC content showed a positive correlation with $q_{max}$ (Table S2). In general, the sorption kinetic process depends on adsorbate properties (size and functional groups), adsorbent texture (pore size), and surface heterogeneity (surface chemistry) [38,50,51]. The degree of heterogeneity has been related to textural properties (porous features) and chemical factors of the sorbent (composition), which may have different implications in the overall sorption process [38], where the higher $k_{2}$ values were observed in small-sized adsorbates by reduction of the MT effects.

### 3.3. Solute Transport Mechanism

#### 3.3.1. Elovich Model

The RAL, MET, and COLL soils presented the highest $R^{2}$ values (0.9890, 0.9794, and 0.9724, respectively; Figure 1c, Table 6). Moreover, the low OC content confers active sites with some characteristics of an energetically heterogeneous surface [22]. The FRU and FRE soils presented the highest values for α and β (Table 6), with a high number of sites available for 2,4-D sorption in the initial phase [34]. The linear plot of qt versus lnt in the Elovich linear equation for each soil is shown in Figure 1c. The highest intercept ($\left(1/\beta \right)ln\left(\alpha \beta \right)$) and slope $\left(1/\beta \right)$ were observed in NBR followed by TCO and FRU, indicating the highest amount adsorbed of 2,4-D during the initial fast phase and rate constant during the slow phase of 2,4-D sorption on these soils, respectively. Nonlinear data analysis results (Figure S1b, Table S1) showed a good adjustment for MET and OSN soils ($R^{2}$ = 0.9525 and $R^{2}$ = 0.9157, respectively) lying in zone III (0.1 > $R_{E}$ > 0.02; the curve rises rapidly) (Figure S1b and Table S1).

#### 3.3.2. Intraparticle Diffusion (IPD) Model

The $q_{t}\;\mathrm{vs}.\;t^{1/2}$ plots were multilinear in all soils (Figure 1d). 2,4-D sorption tends to be followed by two differentiated steps (Figure 1d) [44]. For all VADS, external MT (EMT) was the rate-controlling step at the initial period (45 min) of 2,4-D sorption due to the high %fast ads values (Table 6). For all VADS, the first linear section was far from the origin ($C_{1}$ > 0.00; $R^{2}$ > 0.9323; Table 6), except in DIG ($C_{1}$ = 0.00 and highest % slow ads value; Table 6 and Table 7). A gradual 2,4-D sorption stage was accounted in the second linear section, where 2,4-D diffuses slowly through less accessible sites (smaller pores) on VADS until the equilibrium plateau is reached (Figure 1d; Table 6). Easy access to 2,4-D was observed in RAL where 2,4-D diffuses quickly within micropores (highest $k_{int\;2}$, Table 6) due to the wide distribution of internal pore size. For all VADS, a positive correlation was observed between OC and $C_{1}$, %fast ads and $k_{2}$; %Type 1 sites and $k_{2}$; and %Type 1 sites and h (Table S2). These data placed these soils in the third zone (strong initial sorption, $0.5>R_{i}>0.1$) (Figure S1c, Table S1).

#### 3.3.3. Boyd Model

All plots were linear and passed through the origin, except for FRE, STB, RAL, NBR, and FRU (Figure 1e, Table 6). The film diffusion or chemisorption controls the overall sorption rate of 2,4-D in the last soils (homogenous adsorbents) [21,32,37].

#### 3.3.4. Two-Site Non-Equilibrium (TSNE) Model

For all VADS, this model well described the 2,4-D sorption kinetics data ($R^{2}$ > 0.8269; Figure 1f, Table 6). Concentration decay curves showed an initial step where the 2,4-D uptake was fast in FRU soil (highest F and % fast ads values; Figure 1f, Table 6 and Table 7), followed by a second phase in which the uptake steadily increased up to equilibrium conditions. The high OC content in NBR soil increased the diffusion path length (highest $q_{max}$, lowest $k_{2}$ and high % slow ads values; Table 5 and Table 6), resulting in an IOMD [24]. 2,4-D presented the highest $k_{des}$ values in FRE, STB, DIG, and FRU soils. The fast and slow desorption in soils has been attributed to soft carbon ($OC_{S}$; humic/fulvic acids and lipids) and hard carbon ($OC_{H}$; black carbon), respectively (Table S4). Finally, for all VADS, a direct correlation was observed between $k_{int1}$ and $k_{s}$ (Table S2).

The soils constituents such as Al/Fe oxides, SOM, and mineral composition may influence the chemical non-equilibrium of 2,4-D on VADS. The soil texture plays a more critical role in the ionizable herbicide sorption than OC content on sandy soils [52,53], where Al/Fe oxides promote aggregates. The resulting porosity will differentially impact the chemical associations and soils’ physical properties [21,54] Other studies have indicated that ionizable herbicide sorption such as glyphosate in sandy loam soils is dominated by preferential flow mechanisms (physical non-equilibrium) [52,55].

An overview of the reported $k_{1}$ and $k_{2}$ values obtained in different experimental conditions (contact time, pH and temperature) of 2,4-D on variable-charge soils (VChS), permanent-charge soils (PChS), and different minerals are given in Table S3. The sorbent varied considerably in composition (ranging from pure minerals to soils with different texture, OC content, and mineralogy. These studies showed that 2,4-D sorption kinetics in Andisols followed two-step sorption, and the sorption rate was pH-dependent (Table S3). The first stage accounts for a fast step where most of the sorption occurs, followed by a slower stage (DIP). It was found that chemisorption and external diffusion are the predominant 2,4-D sorption mechanisms.

### 3.4. Sorption Models

2,4-D sorption was studied over a wide range of concentrations (5–25 μg mL^−1^). Table 3 shows a theoretical and empirical description of each sorption model. For all soils, the Freundlich model described the 2,4-D sorption data with $R^{2}$ ≥ 0.9908 (Table 7). 2,4-D was adsorbed in all VADS with $K_{f}$ values between 1.1 and 24.1 µg^1−1/n^ mL^1/n^g^−1^ (Table 7). The highest 2,4-D $K_{f}$ values were observed in NBR, STB, and RAL soils due to their high OC content, Fe/Al oxides, allophane, and positive surface charge (Table 4). Fe/Al oxides, goethite, and gibbsite are also present in these VADS as extractable or free Fe/Al oxides (Table 4) [56]. As both oxides present a IEP value > 7 [57], the functional groups will be found as cationic species at the natural soil pH and would favor the ionic interaction with anionic 2,4-D. In our present work, the pH of Andisols would favor the electrostatic interaction of 2,4-D-SOM because a fraction of neutral species will be present. For Ultisols, the unfavorable conditions for 2,4-D sorption was due to the higher negative net charge (Table 4).

The 1nfads values were lower than one on the MET, FRE, STB, DIG, RAL soils, which correspond to L-type isotherms (Table 3 and Table 7). These results indicate that the 2,4-D sorption first occurred on sorption sites of higher energy, followed by sorption sites of low energy. Table 7 shows a wide range of $K_{foc}$ (17–704), indicating that 2,4-D sorption in the MET, FRE, STB, and RAL soils were not only by means of hydrophobic bonds on OM (highest $K_{foc}$ values, Table 7), but is also related to other inorganic soil constituents (Table 4) [48].

Pesticides with high $K_{oc}$ values (≥1000) have been detected in groundwater and drainage water at several locations worldwide [55], being susceptible to leaching due to its weak sorption on the soil matrix. However, the variability in 2,4-D sorption in VADS is not only associated with SOM, because its soils have variable charge (Al/Fe oxides) than non-VADS [55,58]. In this regard, $K_{oc}$ is not appropriate to describe the potential leaching of 2,4-D in VADS.

For Ultisols, the predominance of more crystalline minerals (higher clay content) and lower OC content (Table 4) likely contributed to the global anionic 2,4-D sorption process. In this regard, if the ratio of mineral to OC fraction is >30, the mineral blockage by OC would be less, allowing the highest mineral contribution (ionic interaction is maximum) [59,60]. The contribution of crystalline clay minerals on Ultisols was significant (ratio between 15.3 to 30.5, Table 4) [48]. The dominant minerals in COLL and MET soils are kaolinite and halloysite, respectively (Table 4). The ionic interaction of 2,4-D in COLL soil should be unfavorable due to the net charge for kaolinite (IEP goes between <3 and 4) at the COLL’s corresponding pH (Table 4).

For DIG soil, the hysteresis coefficient (H) was close to 1 (Table 7), suggesting the 2,4-D reincorporation to the soil solution in this soil. For the other VADS, the markedly irreversible sorption of 2,4-D (H close to 0, Table 7) suggests that 2,4-D reincorporation to the soil solution in VADS must be negligible. The degree of hysteresis depends on various factors related to the pesticides/soil properties and the prevailing conditions [61]. Sorption–desorption hysteresis has been explained as the result of: (i) irreversible binding or sequestration of solute to the OC and/or clay mineral of soil aggregates (binding hysteresis) and (ii) entrapment of sorbed molecules in meso- and microporous structures within mineral structures and OC matrix of soil aggregates (structural hysteresis) [62]. Although it is difficult to identify the relative contribution of binding and structural hysteresis to the total observed hysteresis on VADS, a contribution of both can be observed during 2,4-D desorption from Ultisols and Andisols according to the low $k_{des}$ values.

According to the IPD model for a pesticide, a longer time will take a strongly adsorbed pesticide to achieve the sorption equilibrium than one that is weakly adsorbed [63]. In this regard, 2,4-D presented the lowest $k_{2}$, highest $C_{1}$, and highest $K_{f}$ in RAL and NBR soils, implying that 2,4-D is retained on allophane surfaces and the OC matrix of these soil aggregates (Table 5, Table 6 and Table 7). The longest residence time of 2,4-D on RAL topsoil (lowest $k_{des}$ and $k_{s}$ values; Table 6) could increase its potential to leach into surface or groundwater in this kind of soil (high H value; Table 7). 2,4-D presented the lowest h, highest $t_{1/2}$, and lowest $k_{s}$ values in Ultisols and DIG soil (Table 5 and Table 6). For Ultisols, clay minerals and the lowest OC content contributed to the highest $k_{2}$ values and lowest 2,4-D sorption.

The solute transport process is a function of the soil physical properties, chemical interaction between the solute and soil, and the advective velocity [64]. The 2,4-D transport on VADS should increase if these soils are previously subjected to conventional agriculture (adjustments of $pH_{soil}$ and heavy fertilizer P applications). Andisols exhibit a positive charge under natural environmental conditions, especially when their original pH range is acidic, requiring frequent adjustments of soil pH and heavy fertilizer P applications to remain productive. Our VADS have a strong capacity to retain P, but generally have low availability [21]. Such cases could result in the enhanced mobility of ionizable herbicides because it increases the competition by inorganic anions for positively charged sites and could alter the charge on the oxide surface, changing the speciation [1,23,58,65].

The risk of groundwater contamination would be assumed to be high in fertilized VADS considering conditions of pH alkaline due to the fertilizer P applications, where 2,4-D could strongly compete with fertilizer P for the same sorption sites. In general, weak acids and ionizable molecules dissociate to their anionic form as soil pH increases, which the 2,4-D sorption decreases. In these conditions, anionic 2,4-D will have high mobility in water, quickly reaching aquatic ecosystems. Additionally, this risk would be assumed to be high in VADS subject to minimum tillage (conservation agriculture), increasing the use of herbicides due to the weed pressure. Consequently, the topsoil less disturbed will present: (i) a higher OC content due to the OM accumulation; this accumulation generally acidifies the topsoil, and this condition could increase 2,4-D sorption; and (ii) a higher hydraulic conductivity due to the greater continuity of vertically oriented macropores (transport pores) resulting from the wetting and drying process, root channels, and wormholes. The last point implies that 2,4-D moving down the soil profile should be quick due to preferential flow (nonequilibrium physical).

Therefore, the proper application of agricultural management on VADS is substantial to evaluate the risk of the contamination of groundwater since they will control the amount of herbicide available for vertical transport. In this sense, the 2,4-D sorption mechanism in VADS will depend on the 2,4-D form (ionic or molecular) and the soil physicochemical properties (such as OM, surface charge), where under several external conditions (agricultural management) it could contribute to the sorption of the anionic herbicide on VChS.

An overview of the reported 1nfads, $K_{d}$, $K_{f}$, and $K_{oc}$ values of 2,4-D for VChS and PChS is given in Table S4, showing significant variations in $K_{f}$ values between soils. In general, 2,4-D sorption in VChS and PChS was related to (i) solute parameters such as solubility in water and polarity and (ii) soil parameters such as pH, OM (hydrophobic interactions and hydrogen bonding), and mineral content (hydrophilic mechanism on oxide surfaces through anion exchange) (Table S4). In some soils, pH was found to have a positive correlation with 2,4-D sorption while in others, no correlation was observed. For a significant part of these studies, $K_{d}$ showed a positive correlation with OM content (Table S5). It was found that ligand exchange was the predominant 2,4-D sorption mechanisms in Andisol through the hydroxyl groups from metal–humic complexes and minerals such as allophane (Table S4).

For organic acid, the sorption mechanisms will depend on hydrophobic and hydrophilic sorption domains on VChS [30,66], where these domains will be influenced by the quantity and quality of soil components (minerals and OC). Pesticides can be adsorbed on VADS through mineral (AlSi–Fe) and mineral–organic complexes (AlSi–Fe–HA (humic acids) (through hydroxyl or carbonyl groups of HA from OM) [12]. Regarding the role of soil OM quality, it has been reported that its humic matter fraction can be more important in determining the pesticide sorption parameters given the high reactivity of HA [67]. Additionally, pesticides can be adsorbed by suspended sediment or colloidal matter (dissolved OC (DOC)) enhancing the pesticide leaching [12,68]. Finally, the present study agreed with the results observed in the review tables (Tables S3 and S4), like the presence of two stages of 2,4-D sorption controlled by EMT and found that the kinetic results fit broadly with the PSO model, and the isotherm studies fitted with the Freundlich model.

### 3.5. Exploratory MLR Model of 2,4-D Sorption

**Figure 2:**
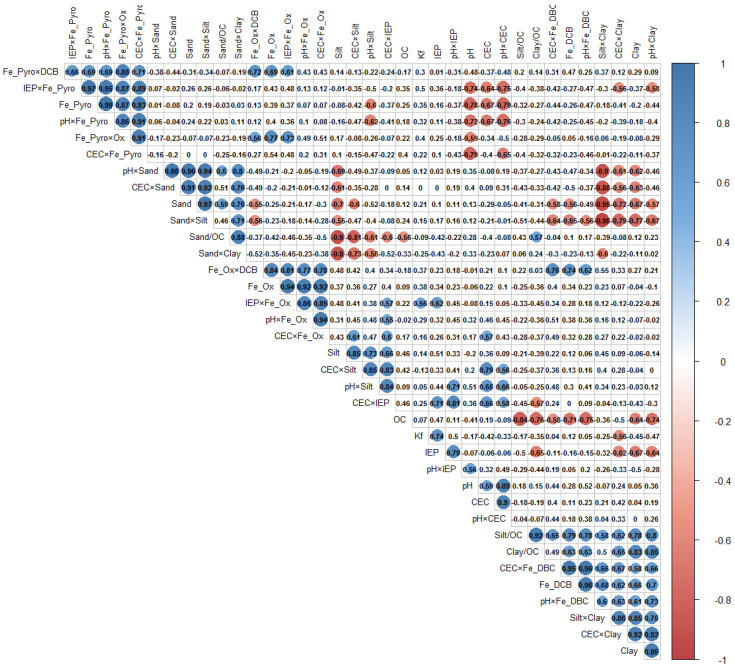
Correlogram of VADS properties and sorption coefficients. Pearson correlation coefficients are proportional to color intensity and the size of the circle.

The 2,4-D $K_{f}$ values for VADS were positively correlated with the IEP of the soils (Figure 2). All the VADS presented an IEP value < 4; the functional groups will be found as anionic species at the pH of each soil and disfavor the ionic interaction with anionic 2,4-D. In other words, IEP values close to soil pH tend to favor the 2,4-D sorption in VADS. Furthermore, in these soils, IEP negatively correlated with clay and clay/OC content. Different relationships between texture soil properties and soil constituents such as Fe oxides content were observed for VADS. For instance, $Fe_{DCB}$ contents were significantly positively correlated with clay, clay/OC, and silt/OC, while those were negatively correlated with sand. Moreover, $Fe_{PYRO}$ contents were significantly negatively correlated with CEC and pH values. For VADS, the 2,4-D $K_{oc}$ values were positively correlated with $Fe_{DCB}$ contents. Both the soil texture and the soil constituents can play an essential role in 2,4-D sorption in VADS.

Different linear combinations of the soil physicochemical properties were evaluated to explain the 2,4-D sorption in VADS (Table S5). In this regard, soil descriptors or pairwise soil interactions (IEP, $Fe_{DCB}$, $Fe_{Ox}$, $IEP\times Fe_{Pyro}$, Sand×Clay, $pH\times Fe_{DCB}$, pH×Silt), with a value of inter-descriptor correlation coefficient lower than 0.6, were selected for the model selection procedures. The pairwise interactions chosen were based on both the outcomes of correlogram of VADS properties and also the properties of soils (e.g., sand and clay interactions moderate the texture of the soil; IEP and $Fe_{PYRO}$ interactions moderate the surface charge of the soil), in order to increase the explanatory power of the model. In the descriptor selection process, the inclusion of pairwise predictor interactions resulted in 2,4-D sorption models with improved fits (Table S5) compared to models that only considered a single variable additive model. The model suggested in this study (Table S5; Equation (1)) is parsimonious and significant with an F-statistic of 8.193, an $R^{2}$ of 0.80, $R_{adj}^{2}$ of 0.71, and the lowest value of AIC, indicating that the model can be used in exploratory terms to assess the importance of soil descriptors on the 2,4-D sorption in VADS. This model can be used as a first approach to obtain a model for predictive purposes. Finally, the chosen subset of significant soil descriptors: IEP, $Fe_{DCB}$, and pH×Silt; represent the most dominant properties of these soils that affect the 2,4-D sorption and explain the 2,4-D sorption mechanism in VADS.

### 3.6. Spectroscopic Analysis

#### 3.6.1. Raman Spectra

**Figure 3:**
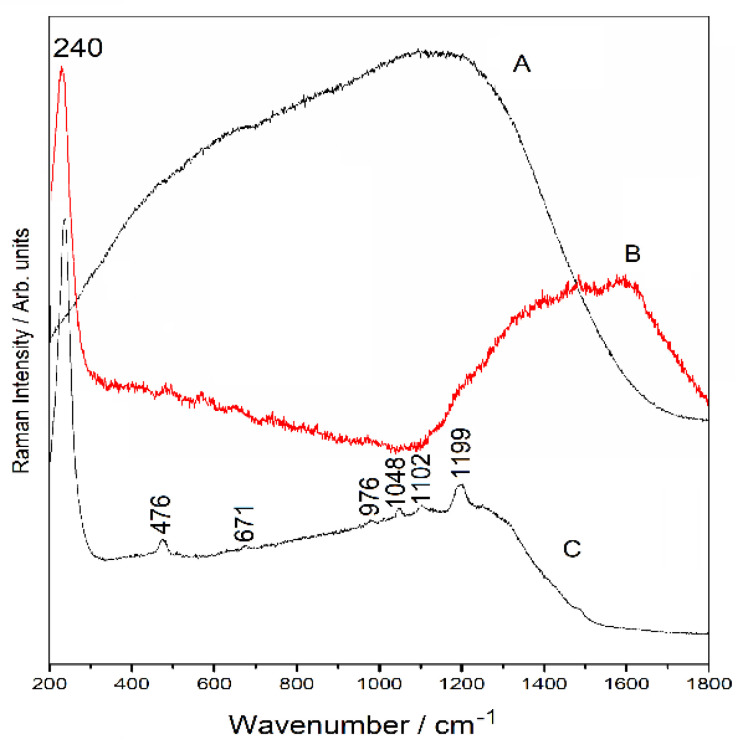
Raman and SERS Spectra of NBR soil sample and system 2,4-D/soil. A, Raman spectrum of NBR soil sample; B, SERS spectra of NBR soil sample; C, SERS spectra of 2,4-D/soil. The same methodology was used to obtain B and C spectra. Laser line 785 was used to record spectrum A and laser line 633 for spectra B and C.

The individual Raman spectra of 2,4-D and the NBR soil sample were recorded (Figure 3). The Raman spectrum of the NBR soil sample did not show important signals, and only a coherent spectral profile could be observed with the high content of OM present in this type of soil (Figure 3). The Raman spectrum for 2,4-D crystals has been fully characterized and assigned. The most probable spectral assignment is presented in Table S6.

#### 3.6.2. SERS Spectra

The interaction of 2,4-D with the NBR soil (Andisol) was evaluated according to the methodology reported here. The corresponding assignment of Raman and SERS bands was carried out first; these assignments are shown in Table S6. The main idea of this analysis was to evaluate and support the previous results regarding how 2,4-D interacts with the NBR soil. In this regard, it is possible to infer an essential interaction between the NBR soil and 2,4-D through the carboxylate group. In the SERS spectrum of 2,4-D interacting with the NBR soil, only signals attributed to the aromatic component of 2,4-D were observed; no signal of the carboxylate group was observed, which allowed us to infer that it is precisely the carboxylate group that holds 2,4-D bonded with the NBR soil (Figure 3). The SERS spectrum of 2,4-D-soil showed bands at 1199, 671, and 476 cm^−1^, which are assigned to CH deformations of the aromatic ring (Figure 3). On the other hand, a set of bands between 976 and 1102 cm^−1^ was mainly attributed to the aromatic ring’s CC and CO stretching modes (Figure 3). The absence of this signal confirms that the carboxylate group directly drives the interaction of 2,4-D with the soil.

## 4. Conclusions

The PSO model well fits the 2,4-D sorption kinetics data on all VADS. Differences in the sorption extent for the fast and slow phases were observed through the IPD model where 2,4-D sorption kinetics was controlled by MT across the boundary layer and IOMD into macro- and micropores in VADS ($C_{1}$ ≠ 0). Consequently, these types of soils provide time-dependent sorption sites (F > 1%). The higher OC content and allophane govern the IPD in Andisols. The presence of kaolinite, halloysite, and Al/Fe oxides govern the IPD in Ultisols. The 2,4-D–VADS systems were classified within the zone of rapidly rising sorption ($0.1>R_{E}>0.02$) and strong initial sorption (Ri<0.4). Equilibrium data, fitted by the Freundlich model, showed that the sorption irreversibility (H ≈ 0) was related to a chemisorption process that controls the initial sorption phase of 2,4-D in VADS. The spectroscopic results support the strong interaction of 2,4-D with the NBR soil, where the carboxylate group keeps it bound to the Andisol soil. This work suggests that the risk for 2,4-D leaching after application on macroporous soils (Andisols) may be increased, as was evidenced by its slow sorption even though 2,4-D is strongly adsorbed. Andisols used in our study are located in regions with high rainfall intensity, conditions that could facilitate its degradation and transport. Taken together, the slow 2,4-D sorption rate on VADS coupled with the low F values highlight the need to consider specific mechanisms and sorption capacity when assessing groundwater contamination from VADS. Further research should focus on models to predict 2,4-D sorption in VADS with regulatory purposes. In this sense, this study allowed for the identification of an exploratory MLR model that emphasizes that IEP, $Fe_{DCB}$, and pH×Silt are important soil descriptors in the 2,4-D sorption in VADS.

## Figures and Tables

**Table 1:** Classification and location of volcanic ash-derived soils used in this study.

Soil	Classification	Location
COLL	Fine, Mesic, Xeric, Paleumult	36°58′ S; 72°09′ W
MET	Fine, Mesic, Paleumult	38°34′ S; 72°22′ W
FRE	Medial, Mesic, Xeric, Placandept	38°57′ S; 72°36′ W
STB	Ashy, Medial, Mesic, Typic, Dystrandept	36°50′ S; 71°55′ W
OSN	Medial, Mesic, Typic, Dystrandept	40°32′ S; 73°05′ W
DIG	Medial, Thermic, Typic, Dytrandept	36°53′ S; 72°10′ W
TCO	Medial, Mesic, Entic Dystrandep	38°6′ S; 72°36′ W
RAL	Mesic, Umbric, Vitrandept	41°32′ S; 73°05′ W
NBR	Ashy, Mesic, Hydric, Dystrandept	41°19′ S; 73°06′ W
FRU	Medial, Isomesic, Typic, Placandept	41°06′ S; 73°07′ W

Republished from [23] with permission from Elsevier Science & Technology Journals. Permission conveyed through Copyright Clearance Center, Inc. COLL, Collipulli; MET, Metrenco; FRE, Freire; STB, Santa Bárbara; OSN, Osorno; DIG, Diguillín; TCO, Temuco; RAL, Ralún; NBR, Nueva Braunau; FRU, Frutillar.

**Table 2:** Uses, properties, and analytical details for 2,4-D.

Pesticide Name (CAS RN)	Molar Mass (g mol^−1^)	Use and APPR ^1^ (g ha^−1^)			${pK}_{a}$ ^2^	$K_{ow}$ ^3^	$S_{w\;}$^4^ (mg L^−1^)		DT_50_^5^ (days)
2,4-D (94-75-7)	221.04	Post-emergence herbicide and selective mode of action. The APPR of 2,4-D is very low (280–2300) for weed control in corn.			2.97	0.027 in alkaline condition, 29.23 in acidic condition	300 at pH= 1 and >20,000 at pH = 5 (20 °C)		10–24
**HPLC Analysis**									
**HPLC mobile phase**		**Flow rate** **(mL min^−1^)**	**Injection volume (µL)**	**Wavelength (nm)**	**Temp** **(°C)**	**Column**		**Detection limit** **(mg L^−1^)**	
65:35 (*v*/*v*) = AcN:water at pH 2.8		1	25	224	35 °C	MultiHigh 100 RP C18 (150 mm × 4.6 mm ID, 5µm).		0.009	

^1^APPR = application rate [29]; ^2^ $pK_{a}$ = dissociation constant; ^3^ $K_{ow}$ = octanol/water partition coefficient [29]; ^4^ $S_{w}$ = solubility in water [30]; ^5^ ${\mathrm{DT}}_{50}$ = half-life time in soil [3].

**Table 3:** Models used to describe sorption kinetic and sorption-desorption of 2,4-D on volcanic ash-derived soils ^1^.

Equation	Equation Number	Parameters	Theoretical and Empirical Description
Adsorbed quantity: $q_{t}=\left(C_{0}-C_{e}\right)\times V/M$	(1)	$q_{t}$: Adsorbed quantity (μg g^−1^) at any soil-solution contact time t (min) for kinetic sorption experiments; $C_{0}$: Initial concentration of 2,4-D in solution; $C_{e}$: Equilibrium concentration of 2,4-D in solution; V/M: Solution/soil ratio.	The adsorbed quantity is obtained from a mass balance between initial and equilibrium concentration of 2,4-D in solution. This equation is valid when degradation and precipitation are negligible during the sorption process.
**Sorption kinetic models**			
Pseudo-first-order (PFO) model [32]: $log\left(q_{max}-q_{t}\right)=logq_{max}-\frac{k_{1}}{2.303}t$	(2)	$q_{max}$: is the maximum sorbed amount (µg g^−1^). $k_{1}$: Rate constant (min^−1^).	This equation fits better at high $C_{0}$ values. The $k_{1}$ is a combination of sorption $\left(k_{a}\right)$ and desorption $\left(k_{b}\right)$ rate constants [33]. Its magnitude is influenced by experimental conditions (pH and temperature) and particle size (small particle size imply large values of $k_{1}$).
Pseudo-second-order (PSO) model [33,34,35,36,37,38,39]: $\frac{t}{q_{t}}=\frac{1}{q_{max}{}^{2}k_{2}}+\frac{1}{q_{max}}t$	(3)	$k_{2}$: Rate constant (g μg^−1^ min^−1^). Derived parameters from Equation (3): h: Initial sorption rate (g μg^−1^ min^−1^), $h=k_{2}q_{max}{}^{2}$; $t_{1/2}$: Half-life time (min), $t_{1/2}=1/\left(k_{2}q_{max}\right)$.	Better fits at low $C_{0}$ values [33]. The $k_{2}$ is a complex function of $C_{0}$ with a time scale factor that decreases when $C_{0}$ increases. Additionally, this model assumes sorption capacity to be proportional to the number of active sites occupied on the soil [40].
**Solute transport mechanism**			
Elovich model [34,41]:		α: Initial sorption rate (μg g^−1^ min^−1^); β: Number of sites available for the sorption (g μg ^−1^), related to the extent of surface coverage and activation energy for chemisorption; $R_{E}=1/\left(q_{ref}\;\beta \right)$: Approaching equilibrium factor. When $R_{E}>0.3$, the curve rises slowly (Zone I), in the range $0.3>R_{E}>0.1$, the curve rises moderately (Zone II); in the range $0.1>R_{E}>0.02$, the curve rises rapidly (Zone III); and when $R_{E}<0.02,$ the curve reaches equilibrium instantly (Zone IV). $t_{ref}$: Longest time in the sorption process ($t_{ref}=t$ at equilibrium); $q_{ref}$: Solid-phase concentration at $t=t_{ref}$ ($q_{ref}=q_{max}$); (*1/β*): rate constant during the slow phase of the reaction.	Describe second order kinetics only for systems with a heterogeneous adsorbing surface. The deviations of the Elovich model at high surface coverage could result in this model neglecting simultaneously occurring desorption. At low surface coverage, this equation might be applied only in cases of strongly heterogeneous surfaces.
$q_{t}=\left(\frac{1}{\beta }\right)ln\left(\alpha \beta \right)+\left(\frac{1}{\beta }\right)ln\left(t\right)$	(4)		
Dimensionless Elovich model [42]:			
$\frac{q_{t}}{q_{ref}}=R_{E}ln\left(\frac{t}{t_{ref}}\right)+1$	(5)		
Intraparticle Diffusion (IPD) model [34,36]:		$k_{int\;i}$: Rate constant of step i (μg g^−1^ min^1/2^); $C_{i}$: Thickness of the boundary layer in step i (μg g^−1^); $R_{i}$: Initial sorption factor in step i (if $q_{ref}=q_{e}$, the applicability of dimensionless *IPD* model is limited to only one step). The initial sorption can be weak (zone I, $1>R_{i}>0.9$), medium (zone II, $0.9>R_{i}>0.5$), strong (zone III, $0.5>R_{i}>0.1$) or complete (zone IV, $R_{i}<0.1$) regarding the equilibrium sorption.	C is proportional to the boundary layer thickness representing the initial sorption on external sites [44]. When $C_{1}=0$, IPD is the most critical rate process controlling sorption; $C_{1}>0$, IPD is not the only rate-controlling step. Thus, the first step must be attributed to the EMT across the boundary layer controlled by liquid film diffusion. The positive intercepts result from the greater boundary layer effect indicating rapid sorption on adsorbents with a wide distribution of pore sizes [43].
$q_{t}=k_{int}t^{1/2}+C$	(6)		
Dimensionless Intraparticle Diffusion (DIPD) model [43]:			
$\frac{q_{t}}{q_{ref}}=1-R_{i}\left[1-{\left(\frac{t}{t_{ref}}\right)}^{\frac{1}{2}}\right]$	(7)		
Boyd model:		$B=-D_{2}\pi^{2}/r^{2}$: Empirical constant related with the effective diffusion coefficient ($D_{2}$) and the effective particle size ($r^{2}$) for the sorption process.	If the plot of Equation (9) is linear with C=0, the rate of mass transfer is controlled by pore diffusion. If the plot is non-linear or linear but C≠0, the film diffusion or chemisorption controls the sorption rate [32].
$\ln\left(1-\frac{q_{t}}{q_{e}}\right)=-0.4977-Bt$	(8)		
Bt=C+k×t	(9)		
Two-Site Non-Equilibrium (TSNE) model [45]: $\frac{C_{t}}{C_{in}}=\frac{1}{R}+\left(\frac{1}{\beta R}-\frac{1}{R}\right)exp\left[-\left(\frac{k_{des}}{\beta }\right)t\right]$	(10)	$C_{t}$: Solute concentration at any time (μg mL^−1^); $C_{in}$: Initial added solute concentration (μg mL^−1^); R: Retardation factor, proportional to the sorption strength; β: Fraction of retardation for Type 1 sites (where sorption is assumed to be instantaneous); $k_{des}$: First-order desorption rate constant for desorption from the Type 2 sites (where sorption is considered time-dependent) (h^−1^). Derived parameters from Equation (10): K: Linear sorption partition coefficient at equilibrium (mL μg^−1^); $K=\left(R-1\right)\times V/M$; F: Fraction of the total sorption in the Type 1 sites when the system is in equilibrium, $F=\left(\beta R-1\right)/\left(R-1\right)$; $k_{s}$: Rate constant for EMT, calculated from the slope of linearisation in the plot of $C/C_{in}$ vs. time t at initial time intervals.	The sorption parameters $k_{des}$ and K are inversely related for neutral organic chemicals in soils and sediments [45]. The $k_{des}$ is considered as a parameter that indiscriminately combines several processes, such as intra-OM diffusion and delayed IPD that control MT of sorbate into the OM complex.
**Sorption–desorption process**			
Sorbed and desorbed fraction:		%ads: Sorbed fraction (%); %des: Desorbed fraction (%); $q_{e\;ads}$ and $q_{e\;des}$: 2,4-D adsorbed in equilibrium (μg g^−1^) for sorption and desorption batch experiments, respectively.	The sorbed fraction can be calculated by means of the IPD model if different steps are present during the sorption process.
$\%fast\;ads=100\times \left(C_{1}/q_{e}\right)$	(11)		
%slow ads=100−%fast ads	(12)		
Linear model: $q_{e}=K_{d}\times C_{e}$	(13)	$K_{d}$: Linear soil-solution distribution coefficient. Derived parameters from Equation (13): $K_{oc}\;\left(from\;K_{d}\right)=100\times K_{d}/\%OC$: OC distribution coefficient from $K_{d}$.	The linear model is useful to describe sorption when the process is independent of the solute concentration.
Freundlich model for sorption:		$K_{f}$: Freundlich constant;1/n: Freundlich sorption coefficient. Derived parameters from Equation (14): $K_{foc}$: OC distribution coefficient from $K_{fads}$; $K_{foc}=100\times K_{fads}/\%OC$; H: Hysteresis coefficient for sorption loop; $H=\left(1/n_{des}\right)/\left(1/n_{ads}\right)$.	The Freundlich model assumes a heterogeneous surface [46]. The single $K_{f}$ term implies that the energy of sorption on a homogeneous surface is independent of surface coverage [47]. In this sense, the energy of binding is the same for the adsorptive sites, and interactions between adsorbed atoms do not exist [46]. The n coefficient is related to the surface heterogeneity and the diversity of the energies associates with the sorption reaction [48]. If 1/n>1, the sorption process shows cooperative sorption; If 1/n=1, *Freundlich* model is equivalent to Linear model indicating low heterogeneity among the sites of the sorbent [48]; If 1/n<1, the relative sorption decreases when the concentration increases. This is characteristic of an L-type sorption isotherm and suggests that specific sites approached saturation as herbicide concentration increased [5], indicating that the sorption firstly occurred on higher energy sites of sorption, followed by low energy sites [49]. A value of H close to 1 means that hysteresis is absent, while a value of H < 1 indicates that hysteresis takes place.
$q_{e\;ads}=K_{fads}\times C_{e\left(ads\right)}{}^{1/n_{fads}}$	(14)		
Freundlich model for desorption:			
$q_{e\;des}=K_{fdes}\times C_{e\left(des\right)}{}^{1/n_{fdes}}$	(15)		

Republished from [23] with permission from Elsevier Science & Technology Journals. Permission conveyed through Copyright Clearance Center, Inc. ^1^ Goodness-of-fit (higher values of determination coefficients ($R^{2}$), lower standard error (*SE*) for each parameter), the relationship between the theoretical basis for each kinetic sorption model was used as criteria to define the most suitable model to describe 2,4-D sorption kinetics 2,4-D transport mechanisms on VADS. Complementary, the accuracy to predict $q_{max}$ (from pseudo-first-order (PFO) and pseudo-second-order (PSO) models) and $K_{d}$ (from Two-Site Non-Equilibrium (TSNE) model) were used.

**Table 4:** Main physicochemical properties and mineral composition of volcanic ash-derived soils used in this study.

Soils	COLL	MET	FRE	STB	OSN	DIG	TCO	RAL	NBR	FRU
Physicochemical Properties										
OC (%)	1.5	2.3	4.5	5.1	5.1	5.8	6.4	6.9	9.5	11.0
$pH_{H_{2}O\;\left(1:2.5\right)}$	5.2	4.7	4.4	5.7	4.6	6.2	5.4	4.4	4.1	4.1
CEC	8.7	9.3	7.1	10.3	9.8	11.8	12.1	7.1	10.3	9.5
Sand (%)	13.7	8.0	21.3	7.2	10.1	35.5	16.1	47.3	6.2	16.3
Silt (%)	40.7	56.7	54.2	66.5	50.9	45.1	58.2	38.5	66.2	63.9
Clay (%)	45.7	35.3	24.5	26.3	39.1	19.4	25.7	12.9	27.6	19.7
$Fe_{PYRO}$ (%)	0.7	0.8	2.2	0.3	1.4	0.4	0.7	1.8	1.8	1.0
$Fe_{OX}$ (%)	0.9	1.8	2.5	1.9	2.0	1.9	2.2	1.4	3.3	0.6
$Fe_{DCB}$ (%)	6.2	7.1	4.3	5.3	3.0	3.5	3.9	1.4	5.1	0.6
IEP	2.0	2.5	3.1	3.8	2.1	2.6	2.9	3.3	3.3	2.9
**Mineral**										
Allophane			+++++	+++++	+++++	+++++	+++++	+++++	+++++	+++++
α-Cristobalite	+		+		+		++	+	+	+
Chlorite–AL				+				++		
Feldspars					+		+		+	
Ferrihydrite			+			+	+		+	
Gibbsite			++	+			++		++	
Goethite		+								
Halloysite	+	+++++		++	+++	++				+
Kaolinite	+++++									
Montmorillonite								+		
Organo-allophanic			++	+	++	+	++		++	+
Plagioclase					+	++		++		+
Quartz		+	+							
Vermiculite	+			++	+	+				++

Republished from [23] with permission from Elsevier Science & Technology Journals. Permission conveyed through Copyright Clearance Center, Inc. ${\mathrm{Fe}}_{\mathrm{PYRO}}$, ${\mathrm{Fe}}_{\mathrm{OX}}$, and ${\mathrm{Fe}}_{\mathrm{DCB}}$ represent Fe oxides extracted by pyrophosphate, acid ammonium oxalate, and dithionite citrate bicarbonate solutions, respectively. +++++ Represents dominant (> 50%), +++ represents common (5–20%), ++ represents present (1–5%), and + represents trace fraction (< 1%).

**Table 5:** Kinetic parameters predicted from linear analysis of pseudo-second-order model.

Parameters	COLL	MET	FRE	STB	OSN	DIG	TCO	RAL	NBR	FRU
$q_{max}$ (exp.)	9.0	9.5	12.0	15.7	13.5	8.1	13.2	24.5	38.9	14.9
Pseudo-second order										
$q_{max}$ (μg g^−1^) ^a^	9.3	8.9	12.1	15.8	14.0	9.9	13.5	24.9	39.7	14.9
$k_{2}$ (g μg ^−1^ min^−1^) ^a^	1 × 10^−2^	2 × 10^−2^	2 × 10^−2^	2 × 10^−2^	1 × 10^−2^	9 × 10^−3^	1 × 10^−2^	9 × 10^−3^	8 × 10^−3^	5 × 10^−2^
$R^{2}$	0.9992	0.9993	0.9997	0.9997	0.9992	0.9997	0.9996	0.9992	0.9999	1.0000
h (g μg ^−1^ min^−1^) ^a^	1.2	1.4	3.6	5.8	2.4	0.9	2.6	5.6	13.3	11.6
$t_{1/2}$ (min) ^a^	7.7	6.4	3.3	2.7	5.7	11.5	5.1	4.5	3.0	1.3

^a^ |Standard error| ≤ 0.1 in all the parameters.

**Table 6:** Kinetic parameters predicted from the linear analysis of Elovich, Intraparticle Diffusion (IPD), and Boyd models and non-linear analysis for Two-Site Non-Equilibrium model (TSNE).

Parameters	COLL	MET	FRE	STB	OSN	DIG	TCO	RAL	NBR	FRU
$K_{d}$ (exp.)	2.4	2.9	4.3	6.9	5.4	2.3	5.1	10.8	50.4	4.7
Elovich										
α (μg g^−1^ min^−1^)	12.0 ± 0.2 ^a^	32.5 ± 0.2	(1.0 ± 0.3)10^3^	(2.0 ± 0.4)10^4^	52.8 ± 0.3	2.6 ± 0.4	(2.0 ± 0.0)10^2^	(1.0 ± 0.1)10^4^	(3.0 ± 2.3)10^2^	(2.0 ± 0.2)10^9^
β (g μg^−1^)	0.8 ± 0.1	1.0 ± 0.1	1.0 ± 0.1	0.9 ± 0.1	0.6 ± 0.1	0.6 ± 0.1	0.7 ± 0.0	0.6 ± 0.1	0.2 ± 0.6	1.8 ± 0.1
$R^{2}$	0.9724	0.9794	0.9239	0.9016	0.9652	0.9505	0.9652	0.9890	0.7961	0.8956
Intraparticle Diffusion										
$k_{int\;1}$ (μg g^−1^)	0.7 ± 0.0	0.6 ± 0.0	0.8 ± 0.0	1.0 ± 0.0	0.8 ± 0.0	1.4 ± 0.1	0.7 ± 0.0	1.0 ± 0.0	4.8 ± 0.6	0.5 ± 0.0
$C_{1}$ (μg g^−1^)	3.2 ± 0.4	3.8 ± 0.3	6.5 ± 0.2	9.4 ± 0.3	6.6 ± 0.2	0.0 ± 0.4	7.3 ± 0.2	16.4 ± 0.1	12.3 ± 2.3	11.7 ± 0.2
$R^{2}$	0.9360	0.9796	0.9968	0.9828	0.9780	0.9824	0.9862	0.9954	0.9323	0.9675
$k_{int\;2}$ (μg g^−1^)	0.2 ± 0.0	0.1 ± 0.0	0.1 ± 0.0	0.1 ± 0.0	0.1 ± 0.0	0.2 ± 0.0	0.1 ± 0.0	0.4 ± 0.0	0.2 ± 0.0	0.1 ± 0.0
$C_{2}$ (μg g^−1^)	6.7 ± 0.1	6.8 ± 0.1	10.5 ± 0.1	14.1 ± 0.0	12.0 ± 0.4	6.2 ± 0.1	11.5 ± 0.2	19.4 ± 0.2	36.4 ± 0.1	14.1 ± 0.0
$R^{2}$	0.9400	0.9788	0.8878	0.9553	0.6203	0.9649	0.9254	0.9630	0.9324	0.9905
Boyd										
C	−0.2 ± 0.2	−0.6 ± 0.5	1.0 ± 0.1	1.0 ± 0.2	0.1 ± 0.2	0.0 ± 0.1	−0.0 ± 0.2	0.5 ± 0.2	1.3 ± 0.2	1.8 ± 0.1
k (min^−1^)	0.04 ± 0.00	0.05 ± 0.00	0.02 ± 0.00	0.03 ± 0.00	0.04 ± 0.00	0.03 ± 0.00	0.04 ± 0.00	0.03 ± 0.00	0.02 ± 0.00	0.02 ± 0.00
$R^{2}$	0.9498	0.8102	0.9333	0.9005	0.9742	0.9699	0.9411	0.9278	0.8289	0.9366
Two Site Non-Equilibrium										
K (mL g^−1^)	2.2 ± 0.0	2.2 ± 0.0	3.7 ± 0.0	5.6 ± 0.0	5.0 ± 0.0	2.0 ± 0.0	4.6 ± 0.0	6.0 ± 0.0	24.3 ± 0.2	2.8 ± 0.0
F	0.4 ± 0.0	0.4 ± 0.0	0.5 ± 0.0	0.5 ± 0.0	0.4 ± 0.0	0.1 ± 0.0	0.5 ± 0.0	0.6 ± 0.00	0.1 ± 0.0	0.7 ± 0.0
$k_{des}$ (h^−1^)	1.9 ± 0.3	1.9 ± 0.2	3.2 ± 0.5	3.2 ± 0.5	1.3 ± 0.3	2.3 ± 0.0	1.4 ± 0.1	1.0 ± 0.1	1.5 ± 0.4	4.2 ± 0.5
$R^{2}$	0.9712	0.9857	0.9713	0.9715	0.9887	0.9840	0.9948	0.9879	0.9939	0.9774
$k_{s}$ (h^−1^)	0.1 ± 0.1	0.1 ± 0.0	0.2 ± 0.0	0.3 ± 0.0	0.2 ± 0.0	0.3 ± 0.0	0.2 ± 0.0	0.1 ± 0.0	0.6 ± 0.0	0.1 ± 0.0
$R^{2}$	0.8269	0.8868	0.9691	0.9378	0.9257	0.9371	0.9518	0.9126	0.8597	0.9116

^a^ Standard error.

**Table 7:** Sorption/desorption of 2,4-D on volcanic ash-derived soils.

Parameters	COLL	MET	FRE	STB	OSN	DIG	TCO	RAL	NBR	FRU
% ads (%)	53	56	52	54	54	56	70	59	69	71
% fast ads (%)	34	43	54	60	47	8	54	55	31	79
% slow ads (%)	66	57	47	41	53	92	46	45	69	22
Linear										
$K_{d}$ (mL g^−1^)	2.4 + 0.0	0.4 + 0.1	4.0 + 0.2	5.2 + 0.4	5.3 + 0.1	2.4 + 0.1	7.8 + 0.3	9.1 + 0.4	48.8 + 1.5	5.6 + 0.1
$R^{2}$	0.9982	0.8777	0.9753	0.9653	0.9961	0.9908	0.9899	0.9886	0.9925	0.9964
$K_{oc}$	160	19	89	103	105	42	122	132	514	51
Freundlich										
$K_{fads}$ (µg^1−1/n^ mL^1/n^g^−1^)	2.6 + 0.1	16.2 + 0.2	18.6 + 0.4	23.4 + 0.7	4.3 + 0.3	10.6 + 0.3	1.1 + 0.1	24.1 + 0.6	20.2 + 0.8	5.3 + 0.4
1nfads	1.0 + 0.0	0.2 + 0.0	0.5 + 0.0	0.5 + 0.0	1.1 + 0.0	0.6 + 0.0	1.7 + 0.0	0.7 + 0.0	1.5 + 0.0	1.0 + 0.0
$R^{2}$	0.9981	0.9908	0.9981	0.9961	0.9971	0.9982	0.9983	0.9987	0.9983	0.9967
$K_{foc}$	175	704	412	460	84	183	17	350	212	48
Desorption										
$K_{fdes}$ (µg^1−1/n^ mL^1/n^g^−1^)	17.3 + 0.2	21.6 + 0.1	33.4 + 0.4	51.0 + 0.5	19.1 + 0.6	16.3 + 0.2	23.3 + 0.4	1.0 + 1.1	104.4 + 1.5	46.7 + 0.4
1nfdes	0.3 + 0.0	0.1 + 0.0	0.3 + 0.0	0.2 + 0.0	0.5 + 0.0	0.4 + 0.0	0.5 + 0.0	0.4 + 0.0	0.4 + 0.0	0.2 + 0.0
$R^{2}$	0.9983	0.9901	0.9971	0.9921	0.9933	0.9987	0.9975	0.9932	0.9923	0.9963
% des (%)	86	92	80	83	75	83	74	72	41	85
Hysteresis										
H	0.3	0.4	0.5	0.4	0.5	0.7	0.3	0.6	0.3	0.2

## Data Availability

The data that support the findings of this study are available from the corresponding author, upon reasonable request.

## Supplementary Materials

The following are available online at https://www.mdpi.com/article/10.3390/ijerph18126264/s1, Table S1: Kinetic parameters predicted from pseudo-first-order, Dimensionless Elovich and Dimensionless Intraparticle Diffusion models, Table S2: Relationship between kinetic sorption, sorption parameters of 2,4-D and volcanic ash-derived soil properties, Table S3: 2,4-D sorption kinetic studies in variable-charge soils and permanent-charge soils, Table S4: 2,4-D sorption studies in variable-charge soils and permanent-charge soils, Table S5: Exploratory MLR models for 2,4-D sorption on volcanic ash-derived soils, Table S6: Experimental Raman and surface enhanced Raman scattering wavenumbers with relative intensities of 2,4-D and 2,4-D-NBR soil systems and the most probable band assignments, Figure S1: Plot for 2,4-D sorption on volcanic ash-derived soils.
