# Supplementary material for: Study of Sorption Kinetics and Sorption–Desorption Models to Assess the Transport Mechanisms of 2,4-Dichlorophenoxyacetic Acid on Volcanic Soils
Source: Int J Environ Res Public Health. 2021 Jun 9;18(12):6264. doi: 10.3390/ijerph18126264 (PMC8296012; doi:10.3390/ijerph18126264)
Supplement: Supplementary file 1 [file ijerph-18-06264-s001.zip › ijerph-1203939-supplementary.pdf]

International Journal of
Environmental Research and Public Health

MDPI

SUPPLEMENTARY INFORMATION

Table S1. Kinetic parameters predicted from pseudo-first-order, Dimensionless Elovich and Dimensionless Intraparticle Diffusion models.

Parameters	COLL	MET	FRE	STB	OSN	DIG	TCO	RAL	NBR	FRU
Pseudo-First Order										
q_{max} (μg g^{-1})	4.9 ± 0.2 [a]	3.2 ± 0.1	2.8 ± 0.3	2.5 ± 0.2	10.1 ± 0.3	5.0 ± 0.1	5.7 ± 0.2	6.2 ± 0.0	10.6 ± 0.3	1.3 ±0.2
k_1 (min^{-1})	0.1 ± 0.0	0.1 ± 0.0	0.1 ± 0.0	0.1 ± 0.0	0.1 ± 0.0	0.1 ± 0.0	0.1 ± 0.0	0.0 ± 0.0	0.1 ± 0.0	0.0 ± 0.0
R^2	0.9297	0.9589	0.8017	0.7930	0.9067	0.9463	0.9449	0.9922	0.8832	0.8508
Dimensionless Elovich										
R_E	0.1 ± 0.0	0.1 ± 0.0	0.1 ± 0.0	0.0 ± 0.0	0.1 ± 0.0	0.1 ± 0.0	0.1 ± 0.0	0.0 ± 0.0	0.1 ± 0.0	0.0 ± 0.0
R^2	0.7388	0.9525	0.7268	0.7069	0.9157	0.3215	0.8826	0.7489	0.6271	0.6998
Dimensionless Intraparticle Difussion										
R_i (min^{0,5})	0.3 ± 0.1	0.4 ± 0.0	0.2 ± 0.0	0.1 ± 0.0	0.3 ± 0.0	0.2 ± 0.1	0.3 ± 0.0	0.1 ± 0.0	0.2 ± 0.1	0.2 ± 0.0
R^2	0.5414	0.8467	0.5163	0.4931	0.7315	0.1746	0.7060	0.5951	0.4029	0.4029

[a] Standard error.

Table S2. Relationship between kinetic sorption, sorption parameters of 2,4-D and volcanic ash-derived soil properties.

Parameters with positive correlation	R^2	n	p-Value
OC content and q_{max}	0.5090	8	0.0001
OC and C_1	0.4276	10	0.0290
%fast ads and k_2	0.4269	10	0.0013
%Type 1 sites and k_2	0.5165	10	0.0011
%Type 1 sites and h	0.5165	10	0.0049
k_{int1} and k_s	0.9333	10	0.0002

Table S3. 2,4-D sorption kinetic studies in variable-charge soils and permanent-charge soils.

**Sorbent**	**Physicochemical properties**					**Methodology**	**Results**	**Ref.**
	pH	Inorganic soil constituent (%)	Fe/Al [a] (%)	OC [b] (%)	$Clay/Silt/Sand$ (%)	Kinetics studies [c]	Kinetics studies [e]	
Three Andisols and a lime silt soil	H_2O = 5.0–6.1	Allophane (0.4–17.8) Ferrihydrite (0.3–2.5)	Oxa = (0.2–1.4)/(0.2–6.0) Pyr = (0.1–0.8)/(0.1–1.1)	OC_T = 0.3–9.0	(12–35)/-/-	2 g of soil wetted to gravimetric water content of 1.5 g g^{-1}/-/$CaCl_2$/5 min–24 h.	Two sorption stages: first stage, where 80% of the maximum sorption happens within the first hour, followed by the second stage of slow sorption after four hours.	[1]
Andisol: Soil removed of OM and hydroxides; Humic acids	H_2O = 5.8 KCl = 4.5 NaF = 11.2	Allophane + Imogolite = (2.1–5.6)	Pyr = 0.4/1 Oxa = (0.1–1.2)/(0.7–4.5) CBD = (0.1–4.2)/-	OC(Oven-dry basis) = 0.2–7.2	-/-/-	250 mg in 5 mL/4–8/$CaCl_2$/1–32 h.	Sorption velocity negatively correlated with pH (pH between 4.5 and 6.5). The highest sorption happens within the first four hours.	[2]
Nine arable soils	H_2O = 5.3–8.2 KCl = 4.4–8.0			OC 0.7–3.2	(6–42)/(5–49)/(13–90)	1:2/-/$CaCl_2$/-[d]	The slow 2,4-D sorption kinetic was due to a diffusive process mainly. The authors observed a direct correlation between time -OC.	[3]
Six soils from Iraq	H_2O = 7.4–8.0 $CaCl_2$ = 7.2–7.8			OC(Walkley-Black)= 1–4	(15–38)/(23–51)/(2–11)	1:3/-/$CaCl_2$/0.5–120 h.	Fast sorption: k_1 (h^{-1}) = 0.1–2.0; Fast desorption: k_2 (h^{-1}) = 5.0–5.4; Slow sorption: k_3 (h^{-1}) = 9.9E^{-4}–2.4E^{-2}; Slow desorption: k_4 (h^{-1}) = 2E^{-6}–2.1E^{-2}. Significative correlations between OC-k_1(+); OC-k_3(+); pH-k_4(-) and sorption-pH (+). A dual sorption/desorption model was used. This model assumes the existence of slow and fast sorption//desorption. The adjustments were found to be significant until a period of agitation of 48 h. This model does not describe the 2,4-D sorption kinetic in soil with low OC content.	[4]
One Andisol and pure kaolinite. *VChS	H_2O = 6.2~6.8 $H_2O_{kaolinite}$ = 6.2			OC_{soil} = 12 $OC_{kaolinite}$ = 0.1	$Clay + Silt_{soil}$ = 58 $Sand_{soil}$ = 42 $Clay + Silt_{kaolinite}$ = 100	Soil: 1:10/6.5 and 4/NaCl+$CaCl_2$/3–48 h. Kaolinite: 1:10/4/NaCl+$CaCl_2$/1–24 h.	The 2,4-D reached a sorption maximum in kaolinite earlier than soil (1 h and 6 h, respectively). Two desorption stages were observed on Andisol at pH 6.5. First is a fast desorption stage followed by a slow one.	[5]
Soil from Nigeria; Soil removed from Fe/Mn oxides and OC. *VChS	$CaCl_2$ = 6.8–7.3	Mainly components: Fe_2O_3 (hematite), SiO_2 (quartz) and Al_2O_3.	Tot = 181–492 (μmol g^{-1})	OC_T= 3–20		1:200/$CaCl_2$/7/0.03–24 h.	Pseudo-second order: k_2 (g mg^{-1} h^{-1}) = 1.0–8.8 (R^2 = 0.970–0.998); h (mg g^{-1} h^{-1}) = 0.07–0.14 (R^2 = 0.970–0.998); Elovich: α (mg g^{-1} h) = 0.07–0.14; β (g mg^{-1}) = 0.04–0.06 (R^2 = 0.866–0.978). Weber-Morris: k_{id}(mg kg^{-1} h$^{-0.5}$) = 0.03–0.06 (R^2 = 0.648–0.933); Liquid film Diffusion: k_{fd} (h^{-1}) = -1.3–0.2	[6]

						(R^2 = 0.944–0.976). A positive correlation between Fe/Mn/OC remotion and specific surface area. Fast initial transfer to the boundary layer, chemisorption and external diffusion were the predominant mechanisms. The highest k_2 was found when OM was removed, and the lowest was found in the original soil.	
Calcareous soil from Turkey	H_2O = 8.4	Silicon oxide = 2.1<br>Carbonate = 96	OC_T = 1<br>OM_T = 2<br>OC_s = 78<br>OC_h= 22	3/30/67	1:5/Natural/Acetone/5–360 min sorption/desorption kinetics using two concentrations (0 and 100 mg L^{-1}).	Pseudo-first order: $k_{1sorption}$= 1.5E^{-5}–3.1E^{-5} (R^2 ^{2}= 0.978–0.989); $k_{1desorption}$= 7.7E^{-7} (R^2= 0.989–0.991); Pseudo-second oder: $k_{2sorption}$(s^{-1}) = 0.5–1.1; $k_{2desorption}$= 1.3E^{-8}–8.30E^{-8} (R^2 = 0.913–0.927). Two phases of sorption: initial fast sorption followed by a slow phase. Two phases of desorption mechanism, initial fast desorption followed by a slow phase, pointing out the existence of hysteresis. Fast desorption was attributed to OC_s and slow desorption to OC_h.	[7]
Volcanic Soil	H_2O = 5.2		$OM_{(Walkley-Black)}$ = 15	17/43/40	1:10/4–6/$CaCl_2$/15 min-24 h/Effect of urea application and pH modification.	Hyperbolic: q_{max} (mg kg^{-1}) = 5.8–9.2 (R^2 = 0.740–0.874). Pseudo-second order: k_2 (g mg^{-1} h^{-1}) = 120–280; h (mg kg^{-1} h^{-1}) = 11.5–25.0 (R^2 = 0.996–0.999). Elovich= (1/y) lnxy (mgkg^{-1}) = 5.0–8.9; 1/y (mg kg^{-1}) = 0.2–1.4 (R^2 = 0.939–0.995). Weber-Morris: C_1 (mgkg^{-1}) =3.4–8.4; C_2 (mg kg^{-1}) = 4.6–8.9; k_{int1} (mg kg^{-1} $h^{1/2}$) = 0.5–3.2; k_{int2} (mg kg^{-1} $h^{1/2}$) = 0.1–1.1 (R^2 = 0.937–0.997). The k_2 values increased with the urea application. A negative correlation was observed between h-pH and C-pH. The initial fast sorption the first hours at more accessible sorption sites, followed by slower sorption ruled by IPD (k_{int2}) to soil micropores. The sorption velocity was found to be slow. This was associated with the presence of the 2,4-D on its anionic form. IPD did not rule the herbicide sorption.	[8]

Types of Fe/Al: Pyr = Extracted with pyrophosphate (associated with OM); Oxa = Extracted with oxalate (amorphous); CBD = citrate bicarbonate dithionite extracted (free form); Tot = total by sequential extraction.; [a] Fe/Al = Iron/Aluminium; [b] OC= organic carbon; OM = organic matter; OC_T = Total OC; OC_s = Soft OC (humic/fulvic acids and lipids); OC_h= Hard OC (black carbon); [c] Kinetic experimental conditions: soil:solution relation/background electrolyte/contact time/observations; [d] Centrifugation parameters: 130 g of soil sample at –33 kPa, incubated for one and seven days; [e] h = initial sorption velocity (from pseudo-second order model); C_1 and C_2 = thickness of the boundary layer associated with the first and second sorption phase, respectively (from Weber-Morris model); k_i = sorption/desorption velocity constant; α = initial sorption velocity (from Elovich model); β = activation energy for chemisorption (from Elovich model).

Table S4. 2,4-D sorption studies in variable-charge soils and permanent-charge soils.

Sorbent	Physicochemical parameters					Methodology	Results	Ref.
	pH/PZNC	Minerals	Fe/Al (%) [a]	OC or OM (%) [b]	$Clay/Silt/Sand$ (%)	Batch studies [c]	Sorption-desorption studies [g]	
Oxisol from Brazil	$CaCl_2$ = 4.9<br>PZC = 4.1	Kaolinite > Gibbsite >> Hematite ≈ Goethite		OC (Walkley and Black) = 1.6	64/-/-	1:2/2.5–6.5 /$CaCl_2$/24 h	K_d (L kg^{-1}) = <5 – 30; K_{OC} (L kg^{-1}) = < 200 – 1700 (R^2= 0.993). Negative correlation between K_d pH. The authors suggest that the main 2,4-D sorption in soils is through its molecular form by interactions of this with OM. A decrease in the 2,4-D sorption with the removal of OM was observed in all the pH range. Predictive K_d equation as a function of pH: $K_d = \{k' OC^d \times f^d) + (k_{oc}^n \times f^n\} * F_{oc} \times \emptyset_m$. This equation does not limit to VADS. It is proposed that variation in the pH can cause conformational changes to OM, thus influencing sorption.	[9]
Granitic Regosol and Dystrophic Red Latosol soils.	H_2O = 4.6–6.5<br>KCl = 4.1–4.5<br>$CaCl_2$ = 4.3–5.1			OC= 0.2–4.6	9–16/4–4.8/52.8–80	1:1/4.0–7.9/$CaSO_4$/24 h	K_d (L kg^{-1}) = 0.02–2.76 (R^2= 0.823–0.993); K_{OC} (L kg^{-1}) = 5.3–287; K_f (L kg^{-1}) = 0.06–4.21 (R^2= 0.944–0.999). Results were fitted to Freundlich and Linear models. Negative correlations between logK_d-pH and logK_{OC}-pH. The following predictive sorption equation was obtained: $\log K_d = 3.83 - 0.39pH + \log C_{oc}$ (SD =24%).	[10]
Thirteen agronomic soils (cambisol, calcisol and regosol)	H_2O = 5.3–8.2			OM= 1.1–6.0	10.4–53.6/13.8–55.4/5.5–73.5	1:5/-/$CaCl_2$/16 h	$K_{f.ads}$ ($mg^{1-n_f}l^{n_f}kg^{-1}$) = 2.5–8.7. Mean desorption = 49.5%. Results were adjusted to Freundlich model. Positive correlation between $K_{f.ads}$-OM. There was not found a significant correlation between $K_{f.ads}$- pH. This is associated with the fact that the 2,4-D would be found in its anionic form at every pH studied. The following predictive equation was found: $K_f = 0.771\ OM - 0.682\ pH + 5.837$. No significant correlations were found to either desorption-pH or desorption-OM.	[11]
Four Oxisols soils. Three Andosols soils. Two Ultisol and Alfisol soil<br><br>*VChS.	H_2O = 4.7–6.1<br>PZNC= <1- 5.1	Allophane. Quartz (less)- Gibbsite. Kaolinite.	CBD = 1.05–13.3/ 0.185–2.83 [12]	OC= 0.7–9.0	20–82/-/-	1:5–1:20/ Ajusted to natural pH/$CaCl_2$-$Ca(H_2PO_4)$-$CaSO_4$-KH_2PO_4-KCl[d] /24h	K_d (L kg^{-1}) = 0.26–18.5 (R^2>0.96); Log $K_{OC.Ca(H2PO4)}$ (L g^{-1}) = 0.77–2.99. Results were fitted to Linear Model. 2,4-D sorption as a function of the electrolyte: $CaCl_2$ > KCl > $CaSO_4$ > $Ca(H_2PO_4)$ ≈ KH_2PO_4. The 2,4-D adsorbed fractions according to mechanisms: Hydrophilic greater than hydrophobic; Anionic exchange greater than Ca^{+2}-bridge (associated with the presence of kaolinite and quartz).<br>A positive correlation between hydrophilic adsorbed fraction and AEC/CEC rate. Significative correlation	[13]

							between hydrophobic sorption-pH(-) and hydrophobic sorption-K_{ow} (+).	
Three Andisol soils and a loam silt soil	H_2O = 5.0–6.1	Allophane (0.4–17.8)<br>Ferryhydrite (0.3–2.5).	Oxa = (0.16–1.44)/(0.15–6.01)<br>Pyr = (0.05–0.76)/(0.05–1.09)	OC_T= 0.32–9.00	(11.9–34.7)/-/-	2g wetted to gravimetric content of 1.5 g g^{-1}/-/$CaCl_2$/24h	K_f (-) = 0.27–7.20 (R^2 = 0.98–1.0); K_d(L kg^{-1}) = 0.16–5.23 (R^2 = 0.89–0.98). Results were fitted to Freundlich model. The K_d of allophanic soils is higher than that of loam silt soil. Positive correlation between K_d-OC, K_d-CEC, K_d-allophane and K_d-ferrihydrite. No significant correlation with pH was found. The possible presence of hummus-Al complex would diminish the positive charge of allophane, thus decreasing sorption.	[1]
Andisol Soil (removed of OM and metallic hydroxides); humic Acid	H_2O = 5.8<br>KCl = 4.5<br>NaF = 11.2	Allophane + Imogolite (2.1–5.6)	Pyr = 0.4/1<br>Oxa = (0.1–1.2)/(0.7–4.5)<br>CBD = (0.1–4.2)/-	OC = 0.15- 7.19	-/-/-	0.25 g in 5 mL/4–8/$CaCl_2$/4h	A negative correlation between sorption and pH. The weak interaction of 2,4-D with OM is given primarily with metal-OM complexes and not by hydrophobic interactions. Strong interaction with active hydroxyls derived from allophane, imogolite, ferrihydrite, goethite and metal-OM complexes by ligand exchange.	[2]
Nine arable soils	H_2O = 5.3–8.2<br>KCl = 4.4–8.0			OC = 0.0–3.2	(5.6–41.5)/(4.6–48.7)/(12.8–89.8)	1:2/$CaCl_2$/72h batch and centrifugation[e]	$K_{d.b}$(mg L^{-1}) = 0.36–3.08; K_{dc1}(mg L^{-1}) = 0.44–2.11. K_{dc7}(mg L^{-1}) = 0.53–2.88. Through the batch method, higher sorption coefficient values were obtained above a specific sorption level.	[3]
Five soils from Slovakia	H_2O = 5.8–8.4	Illite and Esmectite > Caolinite and Cloryte		OC = 0.5–2.4		1:5/Natural/$CaCl_2$/48 h	K_f ($mg^{1-1/n}L^{1/n}kg^{-1}$) = 0.16–3.03 (R^2= 0.90–0.99); K_{OC} (L kg^{-1}) = 33–192. Results were fitted to Freundlich model. Positive correlation between K_f-OC. According to K_{OC} values, 2,4-D should present medium-high mobility in these soils.	[14]
Six soils from Iraq	H_2O = 7.4–8.0<br>$CaCl_2$ = 7.2–7.8			$OC_{(Walkley\text{-}Black)}$ = 1.0–3.8	14.5–38.0/22.5–50.7/2.0–11.4	1:3/-/$CaCl_2$/48 h	$K_{d.teo}$ = 1.74–40094; $K_{d.exp}$= 0.20–7.89. The $K_{d.teo}$ was calculated through kinetic coefficients obtained in this same study. Significative differences were found between theoretical and experimental sorption associated to the existence of non-equilibrium. The dual velocity model does not describe the kinetic behaviour of the herbicide on soils with low OC. Positive correlations between OC-$K_{d.exp}$ and $K_{d.exp} - k_3$. No significative correlation was found between $K_{d.exp}$-pH. It was found that the low velocity sorption coefficient controlled sorption.	[4]
Five agronomic soils from Spain	KCl = 6.3–8.4			OC = 0.3–1.0	2.5–65.9/2.2–45.8/2.7–92.8	Natural/$CaCl_2$/72 h	K_d(mLg^{-1}) = 0.32–2.49. Positive correlations between K_d-OM; K_d-CO_3^{2-}; K_d-$clay$ (the highest correlation). Negative correlation between K_d-pH.	[15]

Andisol soil<br>*VChS	5.7–6.8	OM = 1.7–19.3	$Silt + Clay$<br>31.1–58.1/<br>-/<br>41.9–64.7	1:2/4.2–7.9/$CaSO_4$ ($NaCl+CaCl_2$)/24–48h	K_d (L kg^{-1}) = 0.3–41.1 (R^2 = 0.93–0.99); K_f = 0.1–52.5 (R^2 = 0.76–0.99). Positive correlation between log (K_d)-SOM. A negative correlation between log (K_d)-pH. The following predictive equation was found: $\log(K_d) = 2.04 - 0.37pH + 0.91 \log (SOM)$ (R^2 = 0.83). This predictive equation is efficient for VADS, but its efficiency is reduced in mineral soils. The CEC also was found to be an influential parameter.	[16]
Two soils from Maryland with different kind of grass thatch.			(8.7–12.8)/<br>(10.2–15.8)/<br>(71.2–81.2)	Modified batch [f].	K_f ($mg^{(1-1/n)} L^{1/n} kg^{-1}$) = 0.35–3.14 ($R^2$= 0.96–0.99). Results were fitted to Freundlich model.	[17]
Eight soils from Canada (Ap horizon at low, medium and high slope and on field depression).	H_2O = 6.0–7.6	OC = 0.7–4.0		1:2/Natural/$CaCl_2$/24 h.<br><br>Chemical and structural characteristic of OM were determined and associated to sorption.	K_d (L kg^{-1}) = 0.56–12.54; K_{OC} (L kg^{-1}) = 76–315. Soils from low slope got higher K_d values than those on high slope. High slope got higher K_{OC} values than field depression at higher free C.<br>Positive correlation between K_d-OC, K_d-$HAC-OH$, K_d-$FAC-OH$, K_d-HUC, K_d-$\varepsilon - 280\ HA - OH$. Positive correlation between K_{OC}-$HAC-OH$, K_{OC}-$\varepsilon - 280\ HA - OH$ and K_{OC}-aromatic-C. Any correlations were found between K_d-functional groups. Possible mechanism: Ligand Exchange between COO- from 2,4-D and surface hydroxyls from de metal-humic complexes.[h]	[18]
Two Canada sites. 591 samples.	6.5–9.4	OC= <0.0–5.4	<0.01–48.65/<br>2.60–76.14/<br><0.01–97.41	1:2/-/$CaCl_2$	K_d (L kg^{-1}) = 0.01–6.78; K_{OC} (L kg^{-1}) = 56–170. Positive correlation between K_d-OC. Negative correlation between K_d-pH. Significative correlation between K_d obtained by NIRS and K_d obtained from pedotransfer equation (R^2 = 0.82–0.90). Pedotransfer equation with the highest R^2: $K_d = 0.54 + 1.05\times(SOC\%) - 0.15\times(pH)$.	[19]
Soils from Malaysia. loam clay and clay	5.82–6.17	OC= 2.1–6.0	39.07–50.26/<br>26.76–28.51/<br>21.23–34.17	1:5/3-10/-/4 h	K_d (mL g^{-1}) = 33.83–18.12, R^2 = 0.98–0.99; K_f = 18.83–31.52; K_{OC} = 569.63–862.5. Higher sorption is associated with greater OC content. Negative correlation between K_d-pH.	[20]
Andisol soil<br><br>*VChS	H_2O =<br>6.2–6.8	OC= 12.3	$Clay + slit$ = 58.1<br>Sand = 41.9<br><br>Sorption was studied in soil and pure kaolinite	1:10/4–6.5/$NaCl+CaCl_2$/24 h.<br><br>Sorption/desorption isotherms.<br>Three consecutive desorption.	$K_{f.ads}$ = 0.87–44.46 (R^2 = 0.99), $K_{f.des}$ = 1.56–82.4 (R^2 = 0.56–0.99), ω = 81–1079. Results were fitted to *Freundlich* model. It was found that Andosol presented higher sorption than kaolinite. Significative correlations to Andosol between $K_{f.ads}$-pH (-); $K_{f.des}$-pH (-); ω-pH (-); ω-initial concentration (-). It is suggested that at lower pH strong interaction occurs through ligand exchange. At higher pH, weaker interactions would happen, such as ionic Exchange. No significative correlations were	[5]

							found to kaolinite, but similar trends to Andosol were found.	
Soils from Nigeria; Soils removed of Mn and Fe oxides and OC<br><br>*VChS	$CaCl_2$ = 6.84–7.28<br><br>PZQ = 5.4–6.7	Main components: Fe_2O_3 (hematite). SiO_2 (quartz) y Al_2O_3	Tot = 181.3–492.3 (μmol g-1)	OC_T = 3.3–20.1		1:200/7/$CaCl_2$/24h.<br><br>Sorption/desorption under oxic and anoxic conditions.<br><br>pH effect was evaluated on a different batch experiment (pH 3–8).	$K_{f.ads}$(mg g^{-1} mg L^{-1}) = 1.002–1.148 (R^2= 0.985–0.998); $K_{d.ads}$ (L kg^{-1}) = 0.326–1.404; Log $K_{OC.ads}$ (mg kg^{-1} (mg L^{-1})$^{-1}$) = 0.91- 3.13; Log $K_{OC.des}$ (mg kg^{-1} (mg L^{-1})$^{-1}$) = 0.67–1.4; Log $K_{d.des}$ (L g^{-1}) = 0.56–1.32; H = 0.415–0.966. Results were fitted to Freundlich and *Lineal* model. L-type isotherms were found. The highest sorption under oxic and anoxic conditions was found in the absence of OM. This is associated with the unblock of sorption sites showing lower desorption. It was found that oxides showed higher relevance than OM on the sorption mechanism through electrostatic interactions. Anoxic conditions diminished sorption and increased desorption. Negative correlations between K_d-OC and K_d-pH.	[6]
Mineral soil from West Achaia				OC_T = 1.4	13/4/83	-/-/-/24 h	K_d (m^{3n} kg^{-n}) = 0.0246. The 2,4-D sorption parameters were estimated by fitting the Freudlich model.	[21]
Calcareous soil from Turkey	H_2O = 8.4	Silicon oxide = 2.1<br>Carbonate= 96		OC_T = 1.0<br>OM_T = 1.7<br>OC_s = 78<br>OC_H = 22	3/30/67	1:5/Natural/Acetone/48 h	K_d (L kg^{-1}) = 0.66; K_f (-) = 2.37. Results were fitted to Freundlich and Linear model. No correlation was found, but it is suggested that OM is relevant to the 2,4-D sorption.	[7]

[a] Types of Fe/Al: Extracted with pyrophosphate (Pyr) (associated with OM); Extracted with oxalated (Oxa) (amorphous); CBD = citrate bicarbonate dithionite extracted (free form); Tot = total by sequential extraction; [b] OC = Organic carbon; OC_T = total organic carbon; OC_s = Soft organic carbon (humic/fulvic acids and lipid); OC_H = hard organic carbon (black carbon); [c] Batch parameters: ratio soil: solution/pH /background electrolyte/agitation time; [d] Electrolytes are associated with the fraction of the soil. and every fraction is associated with and sorption mechanism; [e] Centrifugation method parameters: 130 g of sample with a humidity level of –33 kPa, incubated for 1 and 7 days; [f] Isotherms were determined through column flow and leaching analysis; [g] K_d = Linear sorption coefficient; $K_{d.b}$ = K_d determined through batch; K_{dc1}= determined through centrifugation at the first day of incubation; K_{dc7} = determined through centrifugation at the seventh day of incubation; K_f = Freundlich coefficient; K_{OC} = sorption coefficient normalised to OC. In the case of no "ads" subindices, results shown correspond to sorption and there is no desorption data; [h] HA-OH: Humic acids extracted with NaOH; FA-OH: Fulvic acids extracted with NaOH; HU: humin + inorganic particles; ε-280: molar absorptivity at 280 nm.

Table S5. Exploratory MLR models for 2,4-D sorption on volcanic ash-derived soils.

Equation	Subset Selection Methods	MLR	R^2	R^2_{adj}	AIC	F-test	p-Value
(1)	Stepwise full/ Stepwise fwd	K_f = –20.39820 + 16.72031 (IEP) + 1.96378 (Fe_{DCB}) – 0.08585 ($pH \times Silt$)	0.8038	0.7057	34.4	8.193	0.0153
(2)	Stepwise bwd/ Best Subset Selection	K_f = –19.09927 + 17.32191 (IEP) + 0.40808 ($pH \times Fe_{DCB}$) – 0.09832 ($pH \times Silt$)	0.7997	0.6995	34.6	7.985	0.0162
(3)	Stepwise full/ Stepwise bwd	K_f = –12.478787 + 16.919386 (IEP) + –0.006472 ($Sand \times Clay$) + 0.414213 ($pH \times Fe_{DCB}$) – 0.109229 ($pH \times Silt$)	0.8108	0.6595	36.0	5.358	0.0471
(4)	Stepwise fwd	K_f = –18.9821 + 18.1318 (IEP) – 0.4712 ($IEP \times Fe_{Pyro}$) + 2.0203 (Fe_{DCB}) – 0.1017 ($pH \times Silt$)	0.808	0.6545	36.2	5.262	0.0488
(5)	Best Subset Selection	K_f = –26.5332 + 12.0289 (IEP) + 0.5440 (Fe_{Ox}) + 0.9615 (Fe_{DCB})	0.6053	0.408	41.39	3.068	0.1127

Note: fwd = forward; bwd = backward; R^2_{adj} = adjusted R^2 ; AIC = Akike Information criteria; p-value for soil descriptors: IEP < 0.001, $pH \times Silt$ < 0.01, $pH \times Fe_{DCB}$ < 0.05, Fe_{DCB} < 0.05; n = 10. $Variance\ inflation\ factor\ (VIF)_{Soil\ descriptors} < 2.3$.

Table S6. Experimental Raman and surface enhanced Raman scattering wavenumbers with relative intensities of 2,4-D and 2,4-D-NBR soil systems and the most probable band assignments.

Raman 2,4-D	SERS 2,4-D/ Ag	SERS 2,4-D/adsorbed NBR soil	Most probable bands assignment
1661 w	1650 vw		ν CC (ring)
1591 m	1552 vw		ν_{as} COO^- + ν CC (ring)
1441 s			ν_s COO^- + ω CH_2
1393 vw	1359 w		ω CH_2
1310 w			νCC
1255 s	1204 m	1199 s	δCH (ring)
1159 vs	1135 s		δ CH (ring)
1104 m		1102 m	ν CC (ring) + νCO
1050 w		1048 m	δ CH (ring) + νC-O-C
1024 w	1029 m	976 w	ν CC (ring) + ν CO
892 s	889 m		ν C-COO^-
838 s	808 m		ν C-C
717 s	719 w		ν C-Cl + ring def.
683 w		671 w	Ring def.
645 s	637 m		ν CC (ring) + ν C-Cl
592 vw			Ring def.
456 vw		476 m	Ring def. out of plane
413 s			Ring def. + ν C-Cl

ν, stretching; δ, deformation; ω, wagging; ν_s, symmetric stretching; ν_{as}, antisymmetric stretching; Relative intensity: vs, very strong; s, strong; m, medium; w, weak; vw, very weak. Wavenumbers in cm^{-1}.

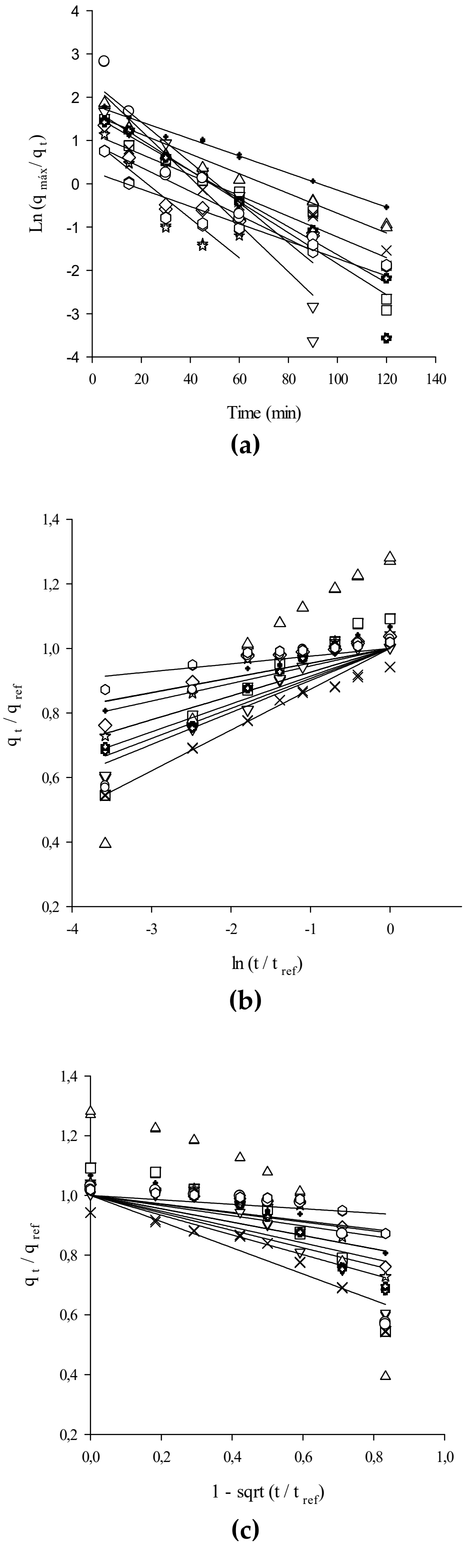

Figure S1. Plot for 2,4-D sorption on volcanic ash-derived soils: COLL (□), MET (×), FRE (☆), STB (◇), OSN (▽), DIG (△), TEM (✚), RAL (+), NBR (○) and FRU (⬡). (**a**) Pseudo-First Order, (**b**) Dimensionless Elovich equation; (**c**) Dimensionless Intraparticle Diffusion Equation. Symbols represent the experimental data, and lines represent the theoretical curves described by each model.

References

1. Duwig, C.; Müller, K.; Vogeler, I.; Mueller, K. 2,4-D Movement in Allophanic Soils from Two Contrasting Climatic Regions. *Commun. Soil Sci. Plant Anal.* **2006**, *37*, 2841–2855, doi:10.1080/00103620600832795.
2. Hiradate, S.; Furubayashi, A.; Uchida, N.; Fujii, Y. Adsorption of 2,4-Dichlorophenoxyacetic Acid by an Andosol. *J. Environ. Qual.* **2007**, *36*, 101–109, doi:10.2134/jeq2005.0415.
3. Kah, M.; Brown, C. Changes in pesticide adsorption with time at high soil to solution ratios. *Chemosphere* **2007**, *68*, 1335–1343, doi:10.1016/j.chemosphere.2007.01.024.
4. Shareef, K.; Shaw, G. Sorption kinetics of 2,4-D and carbaryl in selected agricultural soils of northern Iraq: Application of a dual-rate model. *Chemosphere* **2008**, *72*, 8–15, doi:10.1016/j.chemosphere.2008.02.056.
5. Mon, E.E.; Sharma, A.; Kawamoto, K.; Hamamoto, S.; Komatsu, T.; Hiradate, S.; Moldrup, P. The pH Dependency of 2,4-Dichlorophenoxyacetic Acid Adsorption and Desorption in Andosol and Kaolinite. *Soil Sci.* **2012**, *177*, 12–21, doi:10.1097/ss.0b013e3182376ef3.
6. Ololade, I.A.; Alomaja, F.; Oladoja, N.A.; Ololade, O.O.; Oloye, F.F. Kinetics and isotherm analysis of 2,4-dichlorophenoxyl acetic acid adsorption onto soil components under oxic and anoxic conditions. *J. Environ. Sci. Heal. Part B* **2015**, *50*, 492–503, doi:10.1080/03601234.2015.1018762.
7. Özbay, B.; Akyol, N.H.; Akyol, G.; Ozbay, I. Sorption and desorption behaviours of 2,4-D and glyphosate in calcareous soil from Antalya, Turkey. *Water Environ. J.* **2017**, *32*, 141–148, doi:10.1111/wej.12311.
8. Spuler, M.J.; Briceño, G.; Duprat, F.; Jorquera, M.; Céspedes, C.; Palma, G. Sorption Kinetics of 2,4-D and Diuron Herbicides in a Urea-Fertilized Andisol. *J. Soil Sci. Plant Nutr.* **2019**, *19*, 313–320, doi:10.1007/s42729–019-00031–0.
9. Spadotto, C.A.; Hornsby, A.G. Soil Sorption of Acidic Pesticides. *J. Environ. Qual.* **2003**, *32*, 949–956, doi:10.2134/jeq2003.9490.
10. Nishiguchi, I.; Moldrup, P.; Komatsu, T.; Kodama, Y.; Ito, S. Effect of Organic Carbon Content and pH on Sorption of Linuron and 2,4-D by Three Soils. *Bull. Hiroshima Grad. Sch. Eng.* **2004**, *53*, 15–21.
11. Boivin, A.; Cherrier, R.; Schiavon, M. A comparison of five pesticides adsorption and desorption processes in thirteen contrasting field soils. *Chemosphere* **2005**, *61*, 668–676, doi:10.1016/j.chemosphere.2005.03.024.
12. Hyun, S.; Lee, L.S.; Rao, P.S.C. Significance of Anion Exchange in Pentachlorophenol Sorption by Variable-Charge Soils. *J. Environ. Qual.* **2003**, *32*, 966–976, doi:10.2134/jeq2003.9660.
13. Hyun, S.; Lee, L.S. Quantifying the Contribution of Different Sorption Mechanisms for 2,4-Dichlorophenoxyacetic Acid Sorption by Several Variable-Charge Soils. *Environ. Sci. Technol.* **2005**, *39*, 2522–2528, doi:10.1021/es048820p.
14. Hiller, E.; Krascsenits, Z.; Cernanský, S. Sorption of Acetochlor, Atrazine, 2,4-d, Chlorotoluron, MCPA, and Trifluralin in Six Soils from Slovakia. *Bull. Environ. Contam. Toxicol.* **2008**, *80*, 412–416, doi:10.1007/s00128–008-9430–9.
15. Villaverde, J.; Kah, M.; Brown, C.D. Adsorption and degradation of four acidic herbicides in soils from southern Spain. *Pest Manag. Sci.* **2008**, *64*, 703–710, doi:10.1002/ps.1545.
16. Ei Mon, E.; Hirata, T.; Kawamoto, K.; Hiradate, S.; Komatsu, T. Adsorption of 2,4-Dichlorophenoxyacetic Acid onto Volcanic Ash Soils: Effects of pH and Soil Organic Matter. *Environ. Asia* **2009**, *1*, 1–9.
17. Raturi, S.; Raturi, S.; Hill, R.; Hill, R.; Carroll, M.; Carroll, M. Modeling 2,4-D Transport in Turfgrass, Thatch and Soil. In *Turf Grass: Pesticide Exposure Assessment and Proceedictive Modelings of Tools*; The ACS Symposium Series; American Chemical Society: Washington, DC, USA, 2009; Volume 1028, pp. 157–170.
18. Farenhorst, A.; Saiyed, I.M.; Goh, T.B.; McQueen, P. The important characteristics of soil organic matter affecting 2,4-dichlorophenoxyacetic acid sorption along a catenary sequence. *J. Environ. Sci. Health Part B* **2010**, *45*, 204–213, doi:10.1080/03601231003613542.
19. Singh, B.; Farenhorst, A.; McQueen, R.; Malley, D.F. Near-Infrared Spectroscopy as a Tool for Generating Sorption Input Parameters for Pesticide Fate Modeling. *Soil Sci. Soc. Am. J.* **2016**, *80*, 604–612, doi:10.2136/sssaj2015.03.0118.
20. Ismail, B.; Sameni, M.; Halimah, M. Sorption Kinetics of 2,4-Dichlorophenoxyacetic Acid in Selected Agricultural Soils of Malaysia. *Res. J. Environ. Toxicol.* **2011**, *5*, 39–48, doi:10.3923/rjet.2011.39.48.
21. Magga, Z.; Tzovolou, D.N.; Theodoropoulou, M.A.; Tsakiroglou, C.D. Combining experimental techniques with non-linear numerical models to assess the sorption of pesticides on soils. *J. Contam. Hydrol.* **2012**, *129–130*, 62–69, doi:10.1016/j.jconhyd.2011.09.010.
